# *jouvence*, a new human snoRNA involved in the control of cell proliferation

**DOI:** 10.1186/s12864-020-07197-3

**Published:** 2020-11-23

**Authors:** Flaria El-Khoury, Jérôme Bignon, Jean-René Martin

**Affiliations:** 1 Equipe: Imagerie Cérébrale Fonctionnelle et Comportements (ICFC), Institut des Neurosciences Paris-Saclay (Neuro-PSI), UMR-9197, CNRS/Université Paris-Saclay, 1 Avenue de la Terrasse (Bat. 32/33), 91198 Gif-sur-Yvette, France; 2 Institut de Chimie des Substances Naturelles, CNRS, Université Paris-Saclay, Gif-sur-Yvette, France

**Keywords:** Aging, Cancer, Cell proliferation, Differentiation, RNA-Seq, snoRNA

## Abstract

**Background:**

Small nucleolar RNAs (snoRNAs) are non-coding RNAs that are conserved from archaebacteria to mammals. They are associated in the nucleolus, with proteins to form small nucleolar ribonucleoprotein (snoRNPs). They modify ribosomal RNAs, for example, the H/ACA box that converts uridine to pseudouridine. In humans, various pathologies have been associated with snoRNAs, and several snoRNAs have been reported to participate in many cancer processes. Recently, a new H/ACA box snoRNA named *jouvence* has been identified in *Drosophila* and has been shown to be involved in lifespan determination in relation to gut homeostasis. Because snoRNAs are conserved through evolution, both structurally and functionally, a *jouvence* orthologue has been identified in humans. RT-PCR has revealed that *jouvence* is expressed, suggesting that it might be functional. These results suggest the hypothesis that *jouvence* may display similar functions, including increasing the healthy lifespan in humans.

**Results:**

Here, we report the characterization of the human snoRNA *jouvence*, which has not yet been annotated in the genome. We show that its overexpression significantly stimulates cell proliferation, both in various stable cancerous cell lines as well as in primary cells. By contrast, its knockdown by siRNA leads to the opposite phenotype, a rapid decrease in cell proliferation. Transcriptomic analysis (RNA-Seq) revealed that the overexpression of *jouvence* leads to a dedifferentiation signature of the cells. Conversely, the knockdown of *jouvence* led to a striking decrease in the expression levels of genes involved in ribosome biogenesis and the spliceosome.

**Conclusion:**

The overexpression of a single and short non-coding RNA of 159 nucleotides, the snoRNA-*jouvence*, seems to be sufficient to reorient cells toward stemness, while its depletion blocks cell proliferation. In this context, we speculate that the overexpression of *jouvence*, which appears to be a non-canonical H/ACA snoRNA, could represent a new tool to fight against the deleterious effects of aging, while inversely, its knockdown by siRNA could represent a new approach in cancer therapy.

**Supplementary Information:**

The online version contains supplementary material available at 10.1186/s12864-020-07197-3.

## Background

The snoRNAs are non-coding RNAs, which are conserved from archaebacteria to mammals [1]. They are associated, in the nucleolus, with proteins to form small nucleolar ribonucleoproteins (snoRNPs) [1, 2]. In vertebrates, they are generally processed from introns of pre-mRNAs. Two major classes have been described, C/D box and H/ACA box, based on conserved secondary structures and functional RNA motifs. The C/D box generally performs 2′-O-methylation of ribosomal RNA, while the H/ACA box converts uridine to pseudouridine, and so particularly of ribosomal RNA (rRNA) [1–4]. Moreover, recent studies have shown that the H/ACA snoRNA might also pseudouridinylate other RNA substrates, including mRNA and long-non-coding-RNAs (lncRNAs), and could play a role in chromatin remodelling, suggesting that they could perform several other functions [5]. Furthermore, in humans, various pathologies have been associated with snoRNAs. For instance, a mutation in the H/ACA box of telomerase snoRNA (a RNP reverse transcriptase) gives rise to a pleiotropic genetic disease, congenital dyskeratosis, in which patients have shorter telomeres [6, 7]. Moreover, the snoRNA HBII-52, a human C/D box-type snoRNA, regulates the alternative splicing of the serotonin receptor 2C [8, 9].

In addition to their reported role in some specific pathologies, recent reports suggest that snoRNAs also have tumour-suppressive or oncogenic functions in various cancer types [10]. Indeed, several snoRNAs have been reported to participate in many biological cancer processes, including inactivation of growth suppressors and cell death, activation of invasion and metastasis, and sustained proliferative signalling [11]. Moreover, some reports suggest that snoRNAs could also play a role in cancer stem cells (CSC), as the latter have the capacities of self-renewal, differentiation, and tumorigenicity [12]. Therefore, in this context, snoRNAs could have potential applications for cancer diagnosis and therapy.

In the last few years, in *Drosophila*, we have identified a new small nucleolar RNA (snoRNA) (a non-coding RNA), named *jouvence* (*jou*), the deletion of which reduces lifespan [13]. Inversely, its overexpression increases lifespan. In *Drosophila*, the snoRNA *jouvence* is required in the epithelium of the gut, and more precisely in enterocytes, while its deletion leads to gut hyperplasia/dysplasia in aged flies [13]. Because snoRNAs are well conserved throughout evolution, both structurally and functionally [1–4], we have identified its orthologues in mice and humans. The mouse genome contains two snoRNA *jouvence* genes, whereas only one copy has been identified in humans, located on chromosome 11. RT-PCR showed that, both in mice and humans, *jouvence* orthologues are expressed, suggesting that they might be functional [13]. These results lead to the hypothesis that *jouvence* might display a similar function (increasing healthy lifespan) in mammals, including humans.

Here, we characterized the newly identified snoRNA-*jouvence* in humans, which had not yet been annotated in the genome. First, although the sequence (primary structure) of human-jou shows H/ACA boxes, its predicted secondary structure does not conform to those of typical H/ACA boxes, suggesting that it might be considered a non-canonical H/ACA box snoRNA. Second, we showed, using RT-qPCR, that several cell types expressed the snoRNA-*jouvence*. We also showed that its overexpression importantly stimulates the proliferation of cells, both in immortalized cancerous cells lines (HCT116 and Caco-2) and in non-cancerous cell lines (HEK293 and RPE1), and even in primary cells, such as HUVEC. Conversely, its knockdown by transitory transfection with siRNA leads to the opposite phenotype, inhibiting cell proliferation. Furthermore, a transcriptomic analysis (RNA-Seq) performed on HCT116 overexpressing cells suggests a signature of dedifferentiation of the cells. Finally, a similar transcriptomic analysis performed on the cells in which *jouvence* is knocked-down by siRNA reveals a strong decrease in ribosome biogenesis as well as a decrease of the spliceosome pathways. Therefore, because *jouvence* depletion reduces cancer cell proliferation, we hypothesise that it could represent a good candidate to fight against cancer.

## Results

### Structure, expression, and predicted rRNA target of the human snoRNA-*jouvence*

**Fig. 1:**
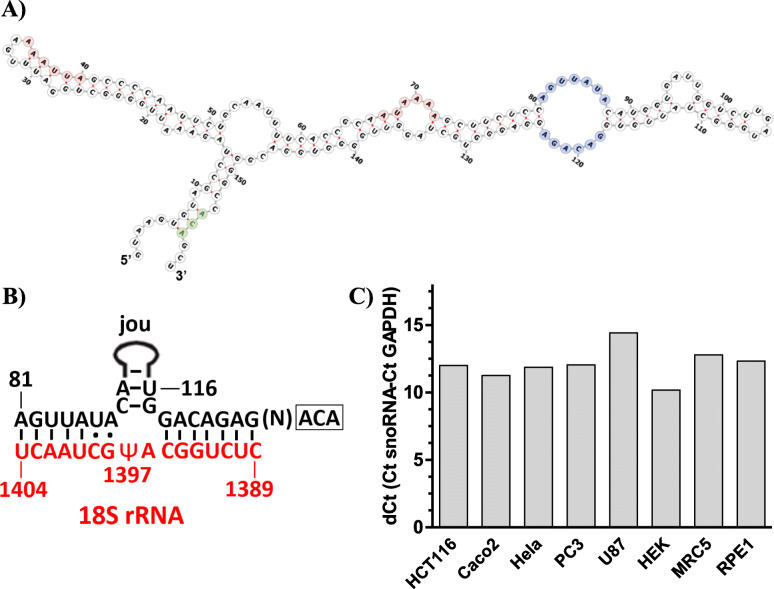
Structure, expression, and rRNA target of the human snoRNA-*jouvence*. **a** Predicted secondary structure of the *h-jou*, as determined by the RNAfold web server (ViennaRNA Web Services, Vienna University, Austria) (http://rna.tbi.univie.ac.at/). Though *h-jou* has a primary sequence in agreement with the H/ACA boxes, its secondary structure does not show a typical double hairpin. Nucleotides highlighted in pink/red correspond to the two putative H (ANANNA) boxes, the nucleotides highlighted in pale-blue are involved in the recognition of the rRNA target, while the nucleotides highlighted in pale-green correspond to the ACA box. **b** Predicted 18S-rRNA target showing the complementary sequences of the two parts of the loop, as well as the putative pseudouridylation site (U in position 1397). **c** Expression level (dCT) (dCT = Ct snoRNA - Ct GAPDH) in various cell lines determined by RT-qPCR (Taqman). Compared to the standard reference gene GAPDH, *h-jou* is weakly expressed in all different tested cell lines, with a very weak expression (to the limit of detection) in the U87 line (glioblastoma), and the strongest expression in the HEK293 cells (human embryonic kidney)

Based on the tertiary (3D) structure of the *Drosophila* snoRNA-*jouvence* determined using the Infernal software (http://eddylab.org/infernal/), we identified the orthologue of *jouvence* in mice and humans, which has not yet been annotated [13]. Human *jouvence* (hereafter named *h-jou*) is located on chromosome 11 (GRCh38.p12) in a long intron of the TEA domain family member 1 gene (named TEAD1) (for the genomic map of the human snoRNA-*jouvence*, see Suppl. Figure 11a in Soulé et al., 2020) [13]. The human genome contains a single copy of *jouvence*, while two copies have been identified in the mouse genome (for the human sequence of the snoRNA-*jouvence*, see Fig. 10 in Soulé et al., 2020) [13]. Because *jouvence* has a primary sequence corresponding to an H/ACA box, its predicted secondary structure was deduced from the ‘RNAfold web server’, and as expected, it is folded to form hairpins (Fig. 1a). However, these complex hairpins do not perfectly conform to the canonical secondary structure of the H/ACA snoRNA, because it does not form two hairpins of similar length, and there is no free terminal ACA box [1, 2]. Nevertheless, the analysis of the internal pocket predicts that it might pseudouridylate the target 18S rRNA on the uridine at position 1397 (Fig. 1b), although this site has never been identified to date as a pseudouridylation site (for a recent pseudouridylation sites determination, see Taoka et al., 2018 [14]). Therefore, we suggest that *h-jou* might be a non-canonical H/ACA box. Furthermore, the expression levels of the snoRNA-*jouvence* was determined using RT-qPCR, in some well-established human cell lines (Fig. 1b). The snoRNA was weakly detected in the nine tested cell lines (first Ct around 30 to 33). Expression levels were similar for all the cell lines, with HEK293 cells expressing a bit more *jouvence* than the other cell lines, with a dCt (Ct snoRNA - Ct reference gene GAPDH) around 10. The U87-MG glioblastoma cells had the lowest expression level with a dCt around 15.

### Overexpression of *h-jou* stimulates cell proliferation

**Fig. 2:**
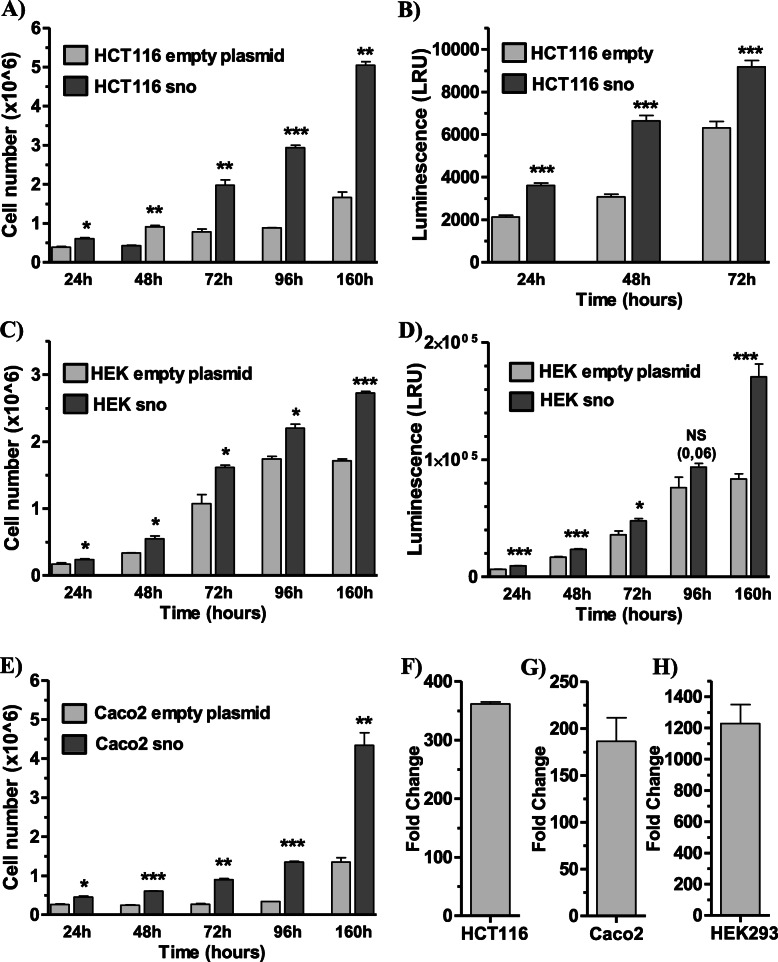
The overexpression of *h-jou* increases the proliferation of the cells. **a** Cells number of HCT116 stably transfected cells with *jouvence* (in plasmid pcDNA-3.1) compared to the empty vector transfected cells determined by VicellXR. **b** Same batch of cells than A, but spread and grow in 96 cell-plates and evaluated by luminescence (CellTiter-Glo), which quantify the amount of ATP, and consequently but indirectly, the number of cells. **c** Cells number of HEK293 stably transfected cells with *jouvence* compared to the empty vector transfected cells determined by VicellXR. **d** Same batch of cells than D, but spread and growth in 96 cell-plates and evaluated by luminescence (CellTiter-Glo). **e** Cells number of Caco-2 stably transfected cells with *jouvence* compared to the empty vector transfected cells determined by VicellXR. The cells overexpressing *jouvence* are more numerous after 24 h post-seeding, which have doubled their number after 72 h, and again, more than tripled after 160 h. **f**, **g**, **h** Expression level (Fold change) of the transfected snoRNA-*jouvence* (overexpression) compared to non-transfected cells. **f** In HCT116, *h-jou* is increased by about 350-fold. **g** In Caco-2, the expression level of *h-jou* is increased by about 200-fold. **h** In HEK293, the expression level is increased by about 1200-fold. Statistics: For the VicelXR (A-C-D) *n* = 2, for the Luminescence CellTiter-Glo (B-D), *n* = 6. Each figure is representative of three independent experiments. (*p*-values: * *p* < 0,05; ** *p* < 0,005; *** *p* < 0,0005). Errors bars represent the mean +/− S.E.M. (*p*-value were calculated using the one-tail unpaired t-test using GraphPad Prism)

To determine the effect of the *h-jou* on human cell lines, the human snoRNA-*jouvence* (159 bp) was stably transfected into HCT116, Caco-2 and HEK293 cell lines. The proliferation of these cells was compared to the corresponding vector (empty plasmid) stably transfected cells. The proliferation was conducted for a period of nearly 1 week for the different cell lines. Cell counting was performed daily using the trypan blue exclusion with VicellXR and/or quantification of ATP using a CellTiter-Glo assay. For the three tested cell lines, the transfection of the snoRNA-*jouvence* allowed cells to proliferate more rapidly. At 160 h post-seeding, HCT116 *h-jou* transfected cells reached nearly 5 million cells vs only 2 million cells for the HCT116 empty plasmid cells: more than two-fold (counted using ViCellXR) (Fig. 2a). The luminescence assay yielded a similar difference (Fig. 2b), although it was less pronounced. Similar results were obtained for the HEK293 snoRNA-*jouvence* stably transfected cells compared to control cells (Fig. 2c, d) with an increase of about 50% of the cell number. Similarly, the Caco-2 snoRNA-*jouvence* stably transfected cells were also more numerous than the corresponding empty plasmid cells (an increase of about 350%) (Fig. 2e). These findings suggest that the overexpression of the snoRNA-*jouvence* stimulates the proliferation of the stably transfected cells. In parallel, to support these results, the corresponding level of snoRNA was assessed using RT-qPCR. We found that HCT116 transfected cells overexpressed *jou* more than 300-fold compared to vector (empty-plasmid) transfected cells (Fig. 2f). Similarly, Caco-2 *h-jou* transfected cells were enriched with *jou* nearly 200-fold (Fig. 2g), while the HEK293 *h-jou* transfected cells had over a 1000-fold increase compared to control cells (Fig. 2h).

### Overexpression of *jou*vence using Lentivirus also increases cell proliferation

**Fig. 3:**
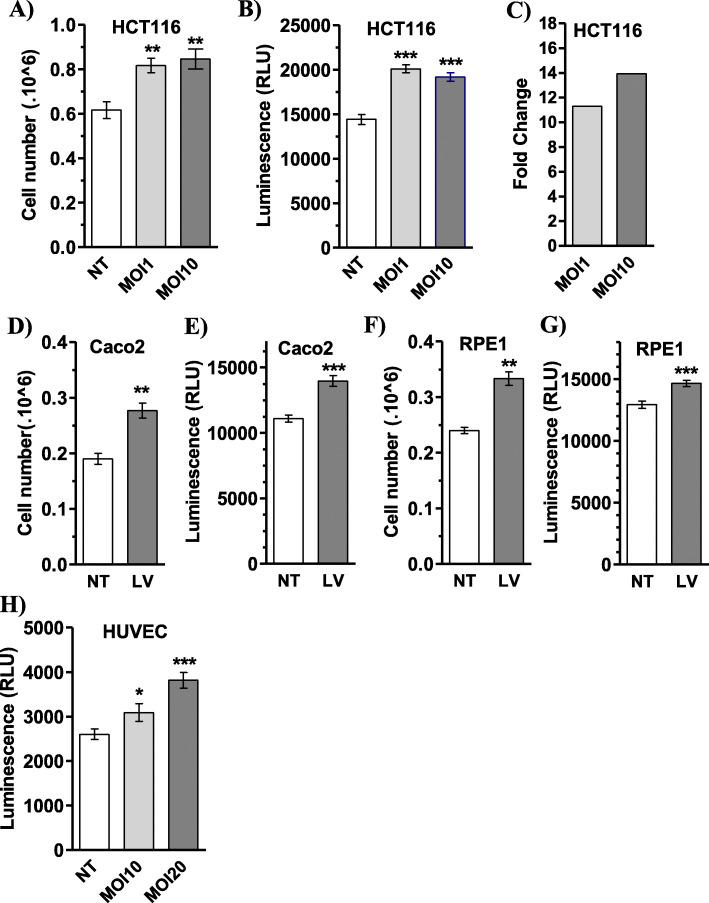
The overexpression of *jou* by lentivirus increases cells proliferation. **a** Cells number of HCT116 cells transduced with *jou*-lentivirus, at two different MOI (1 and 10) compared to non-transduced cells (NT) determined by VicellXR. **b** Same batch of cells than A, but spread and grow in 96 cell-plates and evaluated by luminescence (CellTiter-Glo), which quantify the amount of ATP, and consequently but indirectly, the number of cells. Similarly, the amount of luminescence is increased after 96 h for the two tested MOI. **c** Expression level (Fold change) of the transduced *h-jou* (overexpression) compared to non-transduced cells. In HCT116, *h-jou* is increased by about 11-fold for the MOI-1, and 14-fold for MOI-10. **d** Cells number of Caco-2 cells transduced with *jou*-lentivirus (LV), at MOI-10 compared to non-transduced cells (NT) determined by VicellXR. **e** Same batch of cells than D, but spread and grow in 96 cell-plates and evaluated by luminescence (CellTiter-Glo). **f** Cells number of RPE-1 cells transduced with *jou*-lentivirus (LV), at MOI-10 compared to non-transduced cells (NT) determined by VicellXR. **g** Same batch of cells than F, but spread and grow in 96 cell-plates and evaluated by luminescence (CellTiter-Glo). **h** Cells number of HUVEC transduced with *jou*-lentivirus (LV) at MOI-10 and MOI-20 compared to non-transduced cells (NT), spread and grow in 96 cell-plates and evaluated by luminescence (CellTiter-Glo). The amount of luminescence is increased after 96 h. Statistics: For the VicelXR (A-D-F) *n* = 3, for the Luminescence CellTiter-Glo (B-E-G-H), *n* = 10. For each figure, two independent experiments have been performed. (*p*-values: * *p* < 0,05; ** *p* < 0,005; *** *p* < 0,0005). Errors bars represent the mean +/− S.E.M. (*p*-value were calculated using the one-tail unpaired t-test using GraphPad Prism)

The overexpression of *jouvence* led to an increase in cell proliferation. To date, this effect has only been observed in stably transfected cell lines using plasmid and lipofectamine transfection. However, the constraints of this approach do not easily allow the overexpression of the snoRNA-*jouvence* in primary cells. To study the effect of the overexpression of *h-jou* directly on primary cells, we used the lentivirus approach. After building the lentivirus construct/vector and production of lentiviral particles (see Methods), we transduced the jou-lentivirus into various cells. First, we validated the lentivirus approach in HCT116 and Caco-2 cells to compare the effect on proliferation to the previously observed data obtained in these cell lines. Using two different multiplicities of infection (MOI) (1 and 10), we observed, in HCT116 cells, an increase of cell proliferation determined by two independent readouts (Fig. 3a, b), as previously described with plasmid overexpression. We also measured the level of the snoRNA-*jouvence* using RT-qPCR. Interestingly, we found roughly a 10-fold increase in the amount of *jouvence* (Fig. 3c), with a slight increase according to the MOI. In addition, we also transduced the Caco-2 cell line, and observed an increase in proliferation (Fig. 3d, e). These similar results obtained in two independent cell lines validate the lentivirus approach, and consequently allowed further exploration using the approach on non-cancerous cell lines and on primary cells. Transduction of *jou*-lentivirus into an immortalized but non-cancerous RPE1 cell line (human retinal pigmented epithelial cells) led to a similar increase in cell proliferation (Fig. 3f, g). Ultimately, the transduction of *jou*-lentivirus into primary cells, human umbilical vein embryonic cells (HUVEC), also led to an increase in proliferation, as evaluated by the quantification of ATP using a luminescence assay (Fig. 3h). In summary, the overexpression of snoRNA-*jouvence* using the *jou*-lentivirus transduction system/vector also stimulated the proliferation of non-cancerous primary cells.

### Decreasing *jouvence* levels by siRNA reduces cell proliferation

**Fig. 4:**
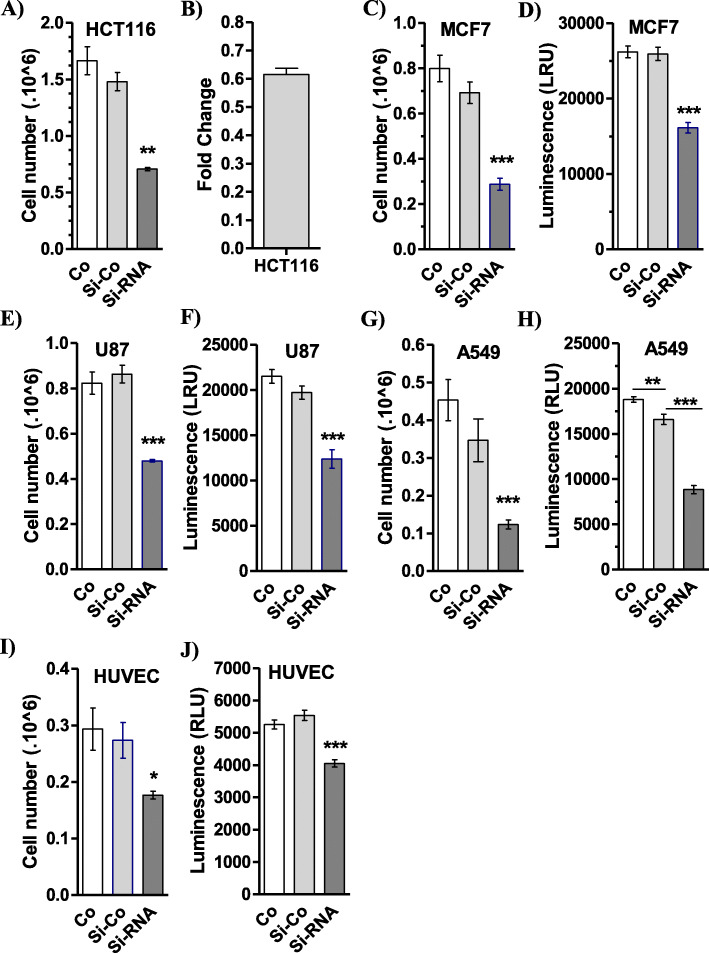
The knockdown of *h-jou* by si-RNA decreases the proliferation. **a** Cells number of HCT116 transiently transfected cells with LNA-siRNA directed against *jouvence* compared to the control non-transfected cells (Co = treated with the same amount of RNAiMAX), and to the control (Si-Co = transfected cells with a siRNA-control without target), determined by VicellXR 72 h post-seeding. **b** The expression level (Fold change) of the siRNA transfected HCT116 cells compared to the non-transfected cells, determined 72 h post-seeding (same batch of cells than A). **c** Cells number of MCF7 transiently transfected cells compared to their two respective controls, as in A, determined by VicellXR. **d** Same batch of cells than C, but spread and growth in 96 cell-plates and evaluated by luminescence (CellTiter-Glo) after 96 h. **e** Cells number of U87 transiently transfected cells with LNA-siRNA compared to their respective controls, determined by VicellXR. **f** Same batch of cells than E, but spread and growth in 96 cell-plates and evaluated by luminescence after 96 h. **g** Cells number of A549 transiently transfected cells with LNA-siRNA compared to their control, determined by VicellXR. **h** Same batch of cells than G, but spread and growth in 96 cell-plates and evaluated by luminescence after 96 h. **i** Cells number of HUVEC (primary cells) transiently transfected cells with LNA-siRNA compared to their respective control, determined by VicellXR. **j** Same batch of cells than I, but spread and growth in 96 cell-plates and evaluated by luminescence. Statistics: For the VicelXR (A-C-E-G-I) *n* = 3, for the Luminescence CellTiter-Glo (B-D: *n* = 27), (H: *n* = 8), (J: *n* = 10). For each figure, two independent experiments have been performed. (*p*-values: * *p* < 0,05; ** *p* < 0,005; *** *p* < 0,0005). Errors bars represent the mean +/− S.E.M. (*p*-value were calculated using the one-tail unpaired t-test using GraphPad Prism)

To determine whether the decrease in the amount of snoRNA-*jouvence* would lead to a modified phenotype, *h-jou* was knocked-down by transiently transfecting an *h-jou* specific siRNA into the HCT116 adenocarcinoma cell line. Briefly, a double siRNA transfection was performed (see Methods for details). The number of cells was assessed after 72 or 96 h depending on the tested cell lines. The negative siRNA mismatch control was also performed under the same conditions. The HCT116 transfected cells with jou-siRNA proliferated at a lower rate compared to their respective controls (non-transfected cells, but treated with the lipofectamine/RNAiMax only [Co], or treated with the negative si-RNA control [si-Co]) with a higher difference seen at 72 h post-transfection (Fig. 4a). The decrease in the amount of specific snoRNA by the siRNA was confirmed by RT-qPCR with a nearly 40% decrease in the endogenous snoRNA at 72 h post-transfection (Fig. 4b). Therefore, we conclude that partial inhibition (a decrease of 40%) of snoRNA-*jouvence* was sufficient to decrease cell proliferation. To assess if this cellular effect of the decrease of *h-jou* is not cell type specific, we performed similar experiments on other well-established cell lines. Similarly, striking decreases in cell numbers were observed for MCF7 cells (a breast cancer cell line) (Figs. 4c, d), U87-MG cells (a glioblastoma cell line) (Figs. 4e, f), and the A549 lung cancer cell line when *h-jou* was knocked-down (Figs. 4g, h). Finally, because the four cell types investigated above are all immortalized cancerous cells, we wondered if similar effects can be observed in primary non-cancerous cells. Knock-down of *h-jou* in HUVEC induced a similar decrease in cell proliferation determined both by cell counting ViCellXR (Fig. 4i), and by ATP measurement (CellTiter-Glo) (Fig. 4j). These results obtained in several different cancerous cell lines and in primary cells suggest that a normal and physiological level of *h-jou* is required for proper cell proliferation. Indeed, increasing the level of *h-jou* stimulated proliferation, while inversely decreasing its level using siRNA inhibited cell proliferation. It also suggests that *h-jou* is functional in all these cell lines derived from various organs and tissues, despite the fact that *h-jou* is only expressed at low levels (Fig. 1c).

### HCT116 overexpressing *h-jou* presents a genomic signature of dedifferentiation

**Fig. 5:**
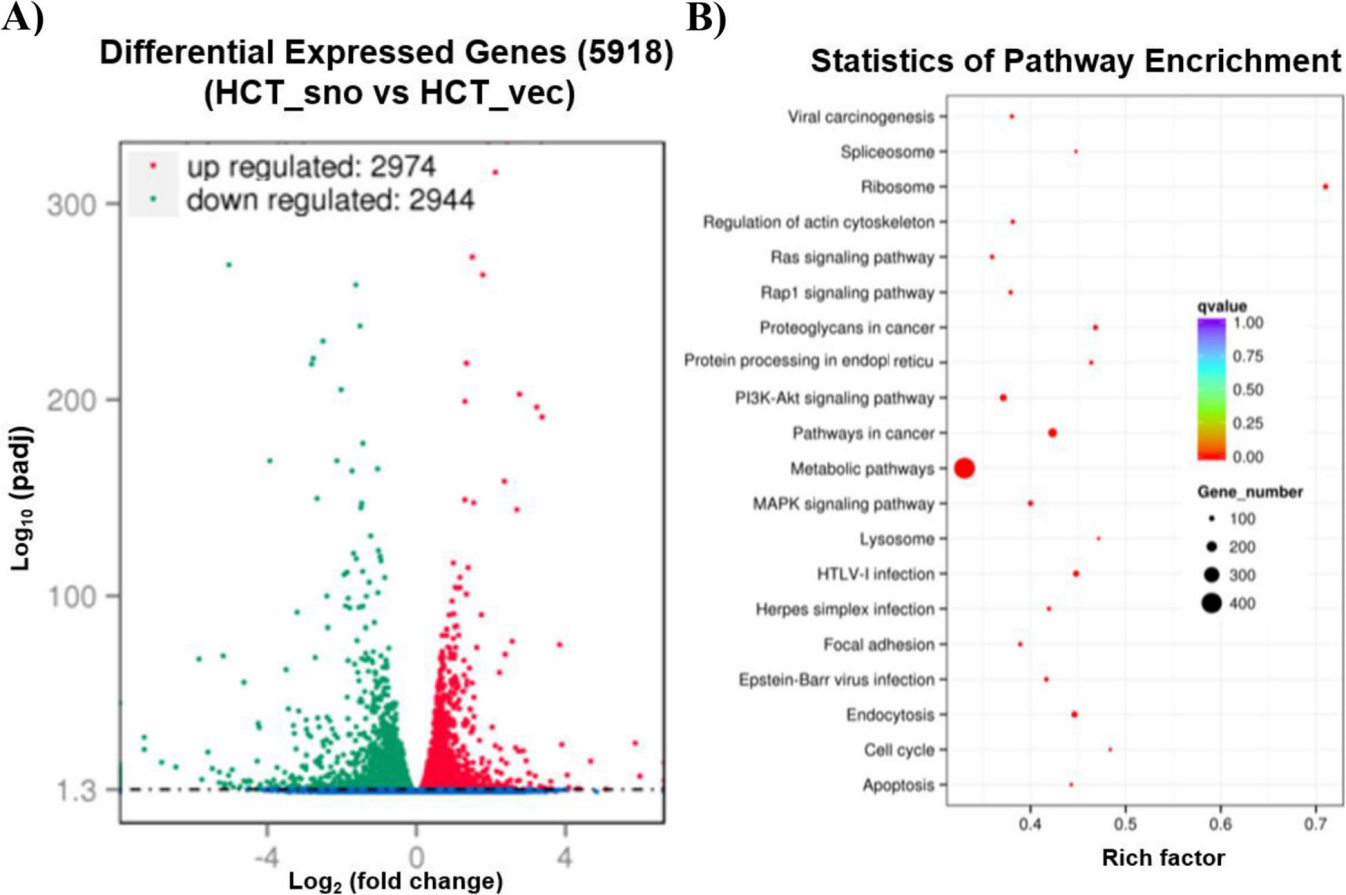
Hundreds of genes are deregulated in HCT116 cells overexpressing *jouvence*. **a** Transcriptomic analysis (RNA-Seq) performed on total-RNA (enriched for poly-A) from the overexpressing *jouvence* HCT116 cells compared to the empty vector cells, reveals that 5918 genes are deregulated, in which 2974 are upregulated, while 2944 are downregulated (see Suppl. Table S1 and Suppl. Table S2 for the complete list of genes). **b** Statistic of enrichment pathway of the deregulated genes according to the KEGG analysis (see Suppl. Table S3) for the full list of KEGG analysis. Rich factor is the ratio of numbers of differentially expressed genes annotated in this pathway term to the numbers of all genes annotated in this pathway term. Greater rich factor means greater intensiveness. Q-value is corrected *P*-value ranging from 0 ∼ 1, with a lower value means greater intensiveness. Top 20 pathway terms enriched are displayed in the figure. In brief, the metabolic pathways are the main deregulated pathways in term of number of genes, while the ribosome is the main deregulated pathway in term of strength (Rich factor)

To elucidate the genetic and molecular mechanisms of snoRNA-*jouvence* that could be responsible for these phenotypes, we first characterized the cells on a whole transcriptomic level. An RNA-seq analysis was performed on HCT116 cells, comparing the snoRNA-*jouvence* stably transfected cells to their empty plasmid controls. The comparison revealed a set of 5918 differentially expressed genes (DEG), with 2974 up-regulated genes and 2944 down-regulated genes (Fig. 5a). Based on the fixed *p*-value, Table 1A lists 20 of the most up-regulated genes (for the complete list of the up-regulated genes, see the Suppl. Table S1, included in: Availability of Data and Materials). Table 1B lists 20 of the most down-regulated ones (see Suppl. Table S2 for the complete list of down-regulated genes, included in: Availability of Data and Materials). To obtain more precise information about these deregulated genes, a statistical enrichment of the DEG in the KEGG pathway [15, 16] showed a significant enrichment in metabolic pathways, with more than 400 DEG with a relatively low rich factor but with high q-values (adjusted *p*-values) (Fig. 5b). Other pathways were also enriched (with high q values), including the RAS signalling pathway, the PI3K-AKT pathway, the regulation of actin cytoskeleton pathway, pathways in cancer, and the ribosome pathway having the highest degree of enrichment, with rich factors of more than 0.7. Again here, based on the p-value, Table 1C lists 18 of the most affected KEGG pathways (see Suppl. Table S3 for the complete list of affected KEGG pathways, included in: Availability of Data and Materials).

**Table 1:** Short list of the main genes deregulated in overexpressing *jouvence* HCT116 cells revealed by RNA-Seq

**Gene_id**	**Fold Change**	**padj**	**Gene Name**	**Description**
**A) HCT116-jou-overexpression (Up-regulated genes)**				
ENSG00000124766	2,82	1,86E-273	SOX4	SRY (sex determ region Y)-box 4
ENSG00000128564	3,43	1,93E-264	VGF	VGF nerve growth factor inducible
ENSG00000049130	2,54	2,33E-219	KITLG	KIT ligand
ENSG00000175745	6,80	1,64E-203	NR2F1	nuclear receptor subfamily 2 group F
ENSG00000158710	2,47	6,78E-200	TAGLN2	transgelin 2
ENSG00000089472	9,35	6,17E-197	HEPH	hephaestin
ENSG00000077274	10,32	7,35E-192	CAPN6	calpain 6
ENSG00000079215	5,15	2,63E-159	SLC1A3	solute carrier family 1 (glial affinity)
ENSG00000127528	2,46	7,89E-150	KLF2	Kruppel-like factor 2 (lung)
ENSG00000146376	2,89	3,19E-148	ARHGAP18	Rho GTPase activating protein 18
ENSG00000165949	6,46	9,82E-145	IFI27	interferon, alpha-inducible protein 27
ENSG00000138434	1,98	1,37E-117	SSFA2	sperm specific antigen 2
ENSG00000120738	2,61	3,23E-115	EGR1	early growth response 1
ENSG00000204574	2,24	3,36E-110	ABCF1	ATP-binding cassette, sub-family F
ENSG00000091136	2,06	4,04E-105	LAMB1	laminin, beta 1
ENSG00000179094	2,25	5,37E-105	PER1	period circadian clock 1
ENSG00000140450	2,13	8,38E-105	ARRDC4	arrestin domain containing 4
ENSG00000115457	2,53	1,12E-101	IGFBP2	insulin-like growth factor bind. prot2
ENSG00000105373	1,94	4,02E-98	GLTSCR2	glioma tumor suppressor
**B) HCT116-jou-overexpression (Down-regulated genes)**				
ENSG00000267761	0,03	1,91E-269	CTD-2130O13.1	–
ENSG00000142910	0,32	2,10E-259	TINAGL1	tubulointerstitial nephritis antigen-like
ENSG00000101188	0,35	2,04E-238	NTSR1	neurotensin receptor 1 (high affinity)
ENSG00000130508	0,18	1,08E-230	PXDN	peroxidasin homolog (Drosophila)
ENSG00000169035	0,15	6,81E-222	KLK7	kallikrein-related peptidase 7
ENSG00000099994	0,14	6,04E-219	SUSD2	sushi domain containing 2
ENSG00000175906	0,25	7,80E-206	ARL4D	ADP-ribosylation factor-like 4D
ENSG00000135074	0,37	1,79E-178	ADAM19	ADAM metallopeptidase domain 19
ENSG00000103044	0,23	9,15E-170	HAS3	hyaluronan synthase 3
ENSG00000108244	0,07	9,31E-170	KRT23	keratin 23 (histone deacet. inducible)
ENSG00000124762	0,49	1,36E-165	CDKN1A	cyclin-dependent kinase inhibitor 1A
ENSG00000101311	0,30	1,40E-164	FERMT1	fermitin family member 1
ENSG00000089356	0,16	1,72E-150	FXYD3	FXYD domain cont. Ion transport
ENSG00000197081	0,36	4,84E-148	IGF2R	insulin-like growth factor 2 receptor
ENSG00000167779	0,36	1,08E-145	IGFBP6	insulin-like growth factor bind. prot6
ENSG00000180921	0,43	2,15E-131	FAM83H	family with sequence similarity 83
ENSG00000117394	0,49	6,20E-124	SLC2A1	solute carrier family 2
ENSG00000148346	0,31	1,58E-122	LCN2	lipocalin 2
ENSG00000130513	0,51	5,91E-121	GDF15	growth differentiation factor 15
**C) HCT116-jou-overexpression (KEGG enrichment pathways)**				
**#Term (KEGG pathway)**	**ID**	**Input number**	**Background number**	***P*-Value-corr**
Ribosome	hsa03010	89	138	3,06E-42
Metabolic pathways	hsa01100	189	1243	8,03E-17
Pathways in cancer	hsa05200	75	397	4,62E-10
Endocytosis	hsa04144	57	260	8,96E-10
Spliceosome	hsa03040	36	134	4,40E-08
Adherens junction	hsa04520	26	74	8,45E-08
HTLV-I infection	hsa05166	51	259	1,47E-07
MAPK signaling pathway	hsa04010	50	255	2,06E-07
Herpes simplex infection	hsa05168	41	186	2,50E-07
RNA transport	hsa03013	38	172	7,33E-07
Epstein-Barr virus infection	hsa05169	42	204	7,33E-07
Tight junction	hsa04530	32	139	3,40E-06
Ras signaling pathway	hsa04014	42	228	8,83E-06
Wnt signaling pathway	hsa04310	31	143	1,39E-05
Huntington’s disease	hsa05016	37	193	1,60E-05
Proteoglycans in cancer	hsa05205	38	205	2,19E-05
Ribosome biogenesis in eukaryotes	hsa03008	23	89	2,50E-05
Protein processing in endoplasmic reticulum	hsa04141	33	166	2,57E-05
Axon guidance	hsa04360	34	176	2,97E-05

A) Up-regulated genes. B) Down-regulated genes. C) Short list of the main deregulated pathways revealed by the KEGG analysis. For the complete list, see Suppl. Tables S1, S2 and S3 respectively

More specifically, amongst the 2974 up-regulated genes, a significant number of them harbouring the best adjusted p-values were found to correlate with dedifferentiation of the cells [17]. Notably, these genes are up-regulated, as in the epithelial-mesenchymal transition (EMT) context, which is controlled by four major interconnected regulatory networks [18–20]. For example, the VGF nerve growth factor inducible (VGF) plays a role in cell plasticity and induces transcription factor TWIST1, which facilitates EMT in cancer cells [21]. The Kit Ligand (KITLG), a ligand for the receptor-type protein-tyrosine kinase, plays an essential role in cell survival, proliferation, haematopoiesis, and stem cell maintenance. KITLG also functions in cell proliferation and adhesion [22]. The nuclear receptor subfamily 2, group F, member 1 (NR2F1), is a nuclear hormone receptor and a transcriptional regulator. It is associated with stem cells, and acquisition of stem-like properties and quiescence [23]. In hypoxic cancer cells, Transgelin 2 (TAGLN2), an actin-binding protein, is induced, while in parallel, Snail1 is increased, leading to the induction of the EMT by downregulating the expression of E-cadherin [22, 24]. The solute carrier family 1 (glial high affinity glutamate transporter), member 3 (SLC1A3), a glutamate transporter, mediates inter-niche stem cell activation [25]. Early growth response 1 (EGR1) factor plays a role in controlling cell plasticity, and has been involved in TGFβ 1-induced EMT [26]. The Kruppel-like factor 2 (KLF2) family are considered to be key transcription factors implicated in self-renewal of embryonic stem cells [27]. All these key genes, amongst others, are considered hallmarks of EMT.

By contrast, 2944 genes were under-expressed. Interestingly, a significant number of these down-regulated genes were shown to be deregulated during EMT, which again suggests that overexpression of the snoRNA-*jouvence* induces EMT. For example, tubulointerstitial nephritis antigen-like 1 (TINAGL1) decreases the secretion of metastasis-suppressive proteins, including insulin-like growth factor binding protein 4 (IGFBP4) [28]. Moreover, it has been reported that EMT in hepatocytes correlates with down-regulation of hepatic differentiation key factors (HNFs) [29]. Here, we found that the expression of HNF4A, the hepatocyte nuclear factor 4α is decreased. Keratin 13 (KRT13) is epigenetically suppressed during transforming growth factor-β 1-induced epithelial-mesenchymal transition [30]. The alpha-like 1 Catenin (CTNNAL1) (a cadherin-associated protein), an epithelial marker, are also under-expressed [31]. Myosin binding protein H (MYBPH) that inhibits cell motility and metastasis is also under-expressed, which is linked to the EMT phenotype [32]. In addition, claudin 9 (CLDN9), an epithelial marker [33], is under-regulated in *h-jou* over-expressing cells, while it was overexpressed in siRNA-transfected HCT116 cells (see below). Annexin 8 (ANXA8), which is also down-regulated, has been shown to be transcriptionally down-regulated by epidermal growth factor (EGF), which correlates with the morphologic changes of EMT [34], along with tumour dedifferentiation. To complete the selected list of markers, the epithelial marker protocadherin 1 (PCDH1) was also down-regulated. PCDH1 binds to SMAD3 and suppresses TGFβ1-induced gene transcription [35].

The overexpressing *h-jou* transfected cells were also enriched in potential cancer stem cell (CSC) markers, including SOX4, SOX8, CD44, MSI-2, EpCAM, and others (Fig. 7). Sox family expression has been correlated with mesenchymal traits and loss of epithelial features. SOX4 plays a role in TGFβ-induced EMT and confers stem cell characteristics [36]. SOX8 regulates CSC properties and EMT via the Wnt β-catenin pathway [37]. The cell surface antigen CD44, Musashi RNA-binding protein 2 (MSI-2) and the Epithelial Cell Adhesion molecule (EpCAM) are also potential CSC markers [38], and were found to be overexpressed in snoRNA-*jouvence*-transfected HCT116 cells. In addition, an important number of KEGG pathways were significantly deregulated (Fig. 5b and Suppl. Table S3), including the insulin secretion pathway, and the insulin signalling pathway. Many of the lipid metabolic pathways were also deregulated, including the glycerophospholipid pathway, glycerolipid metabolism, and the sphingolipid metabolism pathway. The longevity regulating pathways were also affected, with genes such as IRS2, AKT3, IGF1R, NFkB1, FOXO1, and RPTOR. These pathways are known to be linked (directly or indirectly) to EMT, plasticity and longevity of the cells. Finally, to support the RNA-Seq analysis, we also validated, using RT-qPCR, some of the DEG (Suppl. Figure 1A).

### Knockdown of *h-jou* by siRNA decreases ribosome biogenesis and the spliceosome

**Fig. 6:**
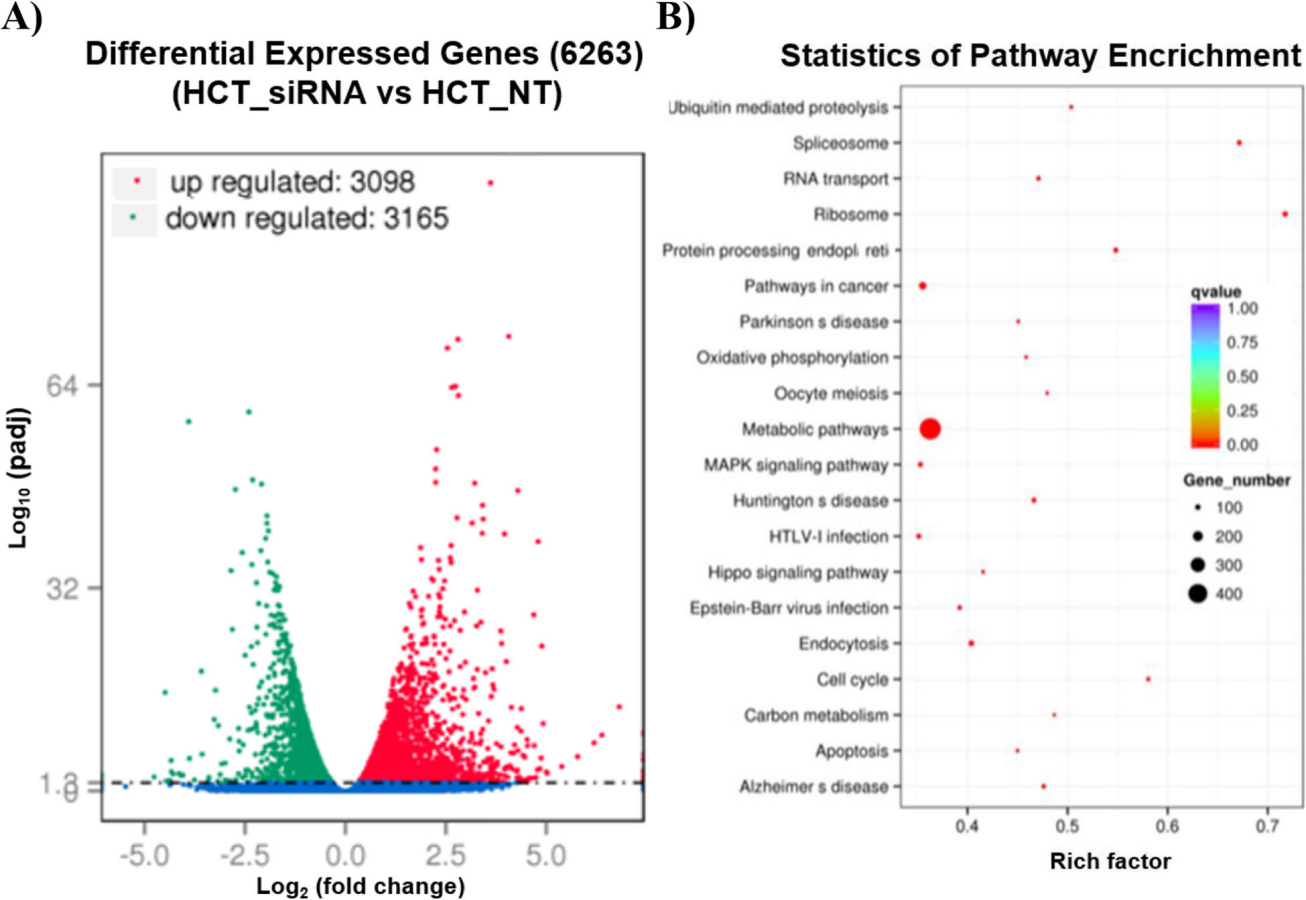
Hundreds of genes are deregulated in *jouvence* depleted HCT116 cells. **a** Transcriptomic analysis (RNA-Seq) performed on total-RNA (enriched for poly-A) from the knockdown of *jouvence* by siRNA on HCT116 cells compared to the control non-transfected cells, reveals that 6263 genes are deregulated, in which 3098 are upregulated, while 3165 are downregulated (see Suppl. Table S4 and Suppl. Table S5 for the complete list of genes). **b** Statistic of pathway enrichment of the deregulated genes according to the KEGG analysis (see Suppl. Table S6 for the full list of the KEGG analysis). In brief, the metabolic pathways are the main deregulated pathways in term of number of genes, while the ribosome and spliceosome are the two main deregulated pathways in term of strength (Rich factor)

Similarly, RNA-seq analysis was performed on HCT116 cells with depletion of *jouvence*. The comparison of the HCT116 *h-jou* knocked-down by siRNA transfected cells compared to the HCT116 non-transfected cells (treated with lipofectamine/RNAiMax only) revealed a set of 6263 DEG, with 3098 up-regulated genes and 3165 down-regulated ones (Fig. 6a). Table 2A lists 20 of the most up-regulated genes (for the complete list, see Suppl. Table S4, included in: Availability of Data and Materials). KEGG analysis pathways presented enrichment in the metabolic pathways, with the highest number of DEG (~ 400) (Fig. 6b). However, the spliceosome and ribosome KEGG pathways had the highest rich factor (around 0.7). Pathways like RNA transport, ubiquitin-mediated proteolysis, protein processing in the endoplasmic reticulum, pathways in cancer, the MAPK signalling pathway, and the hippo pathway, among others, were also enriched with low q values (around 0) and rich factors varying from 0.4 to nearly 0.6.

**Table 2:** Short list of the main genes deregulated in knockdown *jouvence* HCT116 cells revealed by RNA-Seq

**Gene_id**	**Fold Change**	**padj**	**Gene Name**	**Description**
**A) HCT116-jou-knockdown (siRNA) (Up-regulated genes)**				
ENSG00000100867	12,26	1,51E-96	DHRS2	dehydrogenase/reductase (SDR)
ENSG00000151012	16,74	2,25E-72	SLC7A11	solute carrier family 7
ENSG00000164171	6,97	6,83E-72	ITGA2	integrin, alpha 2 (CD49B)
ENSG00000070669	5,81	1,65E-70	ASNS	asparagine synthetase
ENSG00000166750	6,68	1,62E-64	SLFN5	schlafen family member 5
ENSG00000101311	6,26	2,36E-64	FERMT1	fermitin family member 1
ENSG00000205730	7,02	4,60E-63	ITPRIPL2	inositol 1,4,5-trisphosphate recep.
ENSG00000149948	4,82	1,58E-54	HMGA2	high mobility group AT-hook 2
ENSG00000101188	4,75	1,68E-51	NTSR1	neurotensin receptor (high aff.)
ENSG00000179918	4,76	2,44E-49	SEPHS2	selenophosphate synthetase 2
ENSG00000179886	9,29	3,26E-49	TIGD5	tigger transposable element der.5
ENSG00000174564	19,57	4,95E-48	IL20RB	interleukin 20 receptor beta
ENSG00000225614	10,65	9,66E-46	ZNF469	zinc finger protein 469
ENSG00000139178	6,86	9,43E-44	C1RL	complement component 1,
ENSG00000185561	10,75	1,47E-43	TLCD2	TLC domain containing 2
ENSG00000176678	8,90	6,09E-43	FOXL1	forkhead box L1
ENSG00000265688	10,58	2,37E-41	MAFG-AS1	MAFG antisense RNA 1
ENSG00000196922	15,54	3,01E-41	ZNF252P	zinc finger protein 252 pseudoge.
ENSG00000163216	27,76	4,66E-40	SPRR2D	small proline-rich protein 2D
**B) HCT116-jou-Knockdown (siRNA) (Down-regulated genes)**				
ENSG00000142871	0,19	1,84E-60	CYR61	cysteine-rich, angiogenic inducer
ENSG00000186480	0,07	6,02E-59	INSIG1	insulin induced gene 1
ENSG00000079459	0,20	9,36E-50	FDFT1	farnesyl-diphosphate farnesyltr.1
ENSG00000145632	0,24	4,46E-49	PLK2	polo-like kinase 2
ENSG00000114019	0,15	3,07E-48	AMOTL2	angiomotin like 2]
ENSG00000198911	0,26	4,69E-44	SREBF2	sterol regul. Element bind. TF 2
ENSG00000113161	0,26	6,68E-43	HMGCR	3-hydroxy-3-methylglutaryl-CoA red
ENSG00000011426	0,26	9,52E-42	ANLN	anillin, actin binding protein
ENSG00000169991	0,25	1,49E-40	IFFO2	intermediate filament family orphan 2
ENSG00000115963	0,23	1,29E-38	RND3	Rho family GTPase 3
ENSG00000158164	0,17	2,48E-38	TMSB15A	thymosin beta 15a
ENSG00000099860	0,26	8,21E-37	GADD45B	growth arrest and DNA-damage
ENSG00000130164	0,20	2,01E-36	LDLR	low density lipoprotein receptor
ENSG00000052802	0,14	1,79E-35	MSMO1	methylsterol monooxygenase 1
ENSG00000067064	0,27	3,24E-35	IDI1	isopentenyl-diphosphate del iso 1
ENSG00000162772	0,29	5,52E-35	ATF3	activating transcription factor 3
ENSG00000123975	0,30	1,56E-34	CKS2	CDC28 protein kinase regul.sub2
ENSG00000185022	0,30	1,44E-33	MAFF	v-maf musculoaponeurotic fibros.
ENSG00000147642	0,22	1,49E-33	SYBU	syntabulin (syntaxin-interacting)
**C) HCT116-jou-Knockdown (siRNA) (KEGG enrichment pathways)**				
**#Term (KEGG pathway)**	**ID**	**Input number**	**Background number**	***P*-Value-corr**
Metabolic pathways	hsa01100	451	1243	3,20E-50
Ribosome	hsa03010	99	138	5,66E-26
Spliceosome	hsa03040	90	134	2,78E-22
Protein processing in endoplasmic reticulum	hsa04141	91	166	3,31E-18
Cell cycle	hsa04110	72	124	2,03E-15
Pathways in cancer	hsa05200	141	397	6,98E-15
Huntington’s disease	hsa05016	90	193	7,65E-15
Endocytosis	hsa04144	105	260	5,18E-14
Alzheimer’s disease	hsa05010	80	168	1,10E-13
RNA transport	hsa03013	81	172	1,10E-13
Ubiquitin mediated proteolysis	hsa04120	69	137	8,81E-13
Epstein-Barr virus infection	hsa05169	80	204	2,41E-10
Oocyte meiosis	hsa04114	59	123	2,41E-10
Parkinson’s disease	hsa05012	64	142	2,51E-10
Apoptosis	hsa04210	63	140	3,55E-10
Oxidative phosphorylation	hsa00190	61	133	3,75E-10
Carbon metabolism	hsa01200	55	113	5,90E-10
HTLV-I infection	hsa05166	91	259	8,16E-10
MAPK signaling pathway	hsa04010	90	255	8,16E-10

A) Up-regulated genes. B) Down-regulated genes. C) The main deregulated pathways revealed by the KEGG analysis. For the complete list, see Suppl. Tables S4, S5 and S6 respectively

**Fig. 7:**
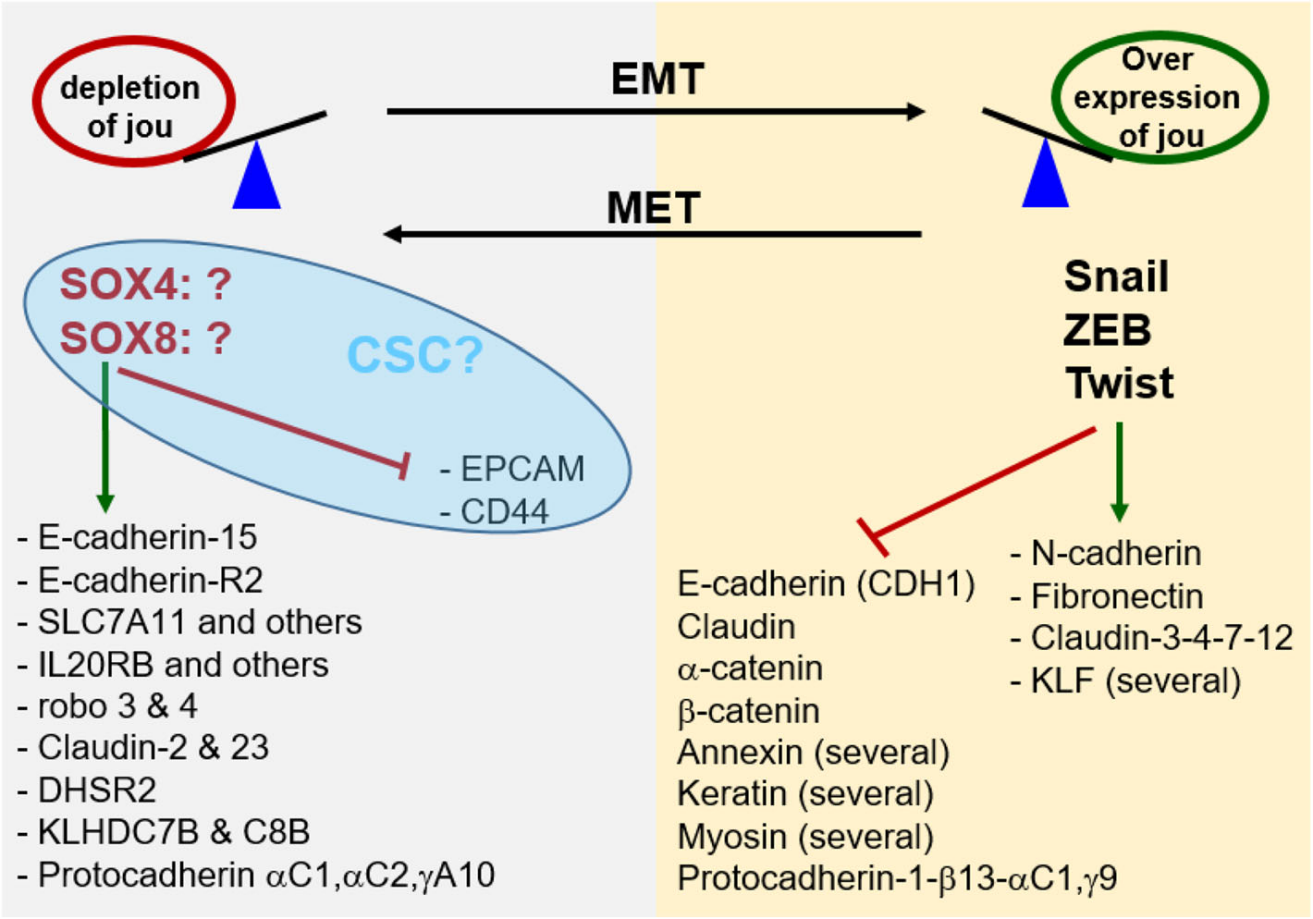
Summary of main deregulated genes suggesting a dedifferentiation. Several of the main key Transcription Factors genes (Twist, Snail, ZEB) involved in EMT are upregulated in *jou*-overexpression. Consequently, several of their known targets as N-cadherin, Fibronectin, few Claudin and several KLF are upregulated. In parallel several genes are downregulated, as E-cadherin (CDH1), Claudin, α-catenin, β-catenin, several Annexin, several Keratin and several Myosin, as well as Protocadherin1-β13, αC1, γ9. Inversely, in the *jou-*depleted cells by siRNA, we rather observe the opposite phenotype, suggesting a MET (Mesenchymal-Epithelial-Transition). More particularly, we observe a decrease of two Transcription Factors as SOX4 and SOX8, although these two last have not yet been clearly demonstrated to induce MET. Several other genes (putatively their targets) are upregulated, while two key genes, as EPCAM and CD44 are downregulated. This last group of four genes (blue ellipse) could also suggests a CSC phenotype. Nevertheless, although we do not observe all the characteristic cells markers of the EMT, several of the deregulated genes in *jou*-overexpression strongly suggest an EMT, or at least a partial or hybrid EMT, as suggested by Pastushenko and Blanpain (2019) [19], while inversely, the knock-down of *jouvence* rather seems to direct the cells toward a MET

More specifically, the solute carrier family 7 (anionic amino acid transporter light chain), member 11 (SLC7A11) and interleukin 20 receptor-β (IL20RB) were upregulated in the in the presence of siRNAs. Kelch domain containing 7B (KLHDC7B), which has been associated with gene modulation activity in the interferon signalling pathway [39], was also upregulated after the snoRNA knock-down (fold-change of 83.3). Drosophila Roundabout homolog 4 (ROBO4), an axon guidance receptor, which increases cell adhesion [40], correlating with an epithelial phenotype, was also upregulated (fold-change of 73.09). Cadherin 15, type 1, M-cadherin (CDH15) and claudin 2 (CLDN2), which are epithelial markers, were overexpressed when the snoRNA was depleted. This suggests that inhibition of snoRNA led to a more pronounced epithelial phenotype (MET) while inversely, its overexpression favoured EMT (Fig. 7).

On the other hand, knockdown of *h-jou* led to down-regulation of 3165 genes, with a majority of down-regulated genes belonging the ribosome or spliceosome pathways. Table 2B lists 20 of the most down-regulated genes (for the complete list, see the Suppl. Table S5, included in: Availability of Data and Materials). Moreover, based on the *p*-value, Table 2C lists 19 of the most affected KEGG pathways (see Suppl. Table S6 for the complete list of affected KEGG pathways, included in: Availability of Data and Materials). This implicates the H/ACA box snoRNAs in ribosome biogenesis, the modification and processing of ribosomal RNA precursors [1–4]. More specifically, splicing factors were down-regulated, including serine/arginine-rich splicing factor 4 (SRSF4), serine/arginine-rich splicing factor 11 (SRSF11), splicing factor 3b, subunit 1, 155 kDa (SF3B1) and splicing factor 3b, subunit 2, 145 kDa (SF3B2) (Suppl. Table S5). Some ribonucleoproteins were also down-regulated: heterogeneous nuclear ribonucleoprotein H1 (HNRNPH1), and small nuclear ribonucleoprotein 70 kDa (U1) (SNRNP70) (Table 2B and Suppl. Table S5). In other words, the main deregulated KEGG pathways (with the highest rich factors) were the ribosome and the spliceosome pathways (Table 2C). For the ribosome pathway, 90 genes were down-regulated over the 99 genes. For the spliceosome pathway, of the 90 total genes, 80 were down-regulated. These effects correlate with the main known function of the snoRNAs in guiding chemical modifications of other RNAs, mainly rRNAs, transfer RNAs and small nuclear RNAs, through their function within their related RNPs. Finally, we also validated, using RT-qPCR, some of these DEG: CLN2, HMGCR, PRPF3, SRSF4, DHRS2, IL20RB, and SLC7A11 (Suppl. Figure 1B).

### Several genes are deregulated in the opposite direction when comparing overexpression and depletion of *jou*

RNA-Seq analysis revealed that several genes are differentially expressed either up or down; i.e., there was both overexpression and knockdown of snoRNA-*jouvence*. We wondered if the genes that were upregulated in the *h-jou* overexpression condition were inversely downregulated in the inverse *jouvence*-knocked-down condition, and vice-versa. When the upregulated genes in the *jouvence*-overexpression condition were compared to the down-regulated genes in the *jouvence* depletion (siRNA condition), we found 1102 DEG in common (see Suppl. Table S7 for the list of genes, included in: Availability of Data and Materials). Conversely, the comparison of the down-regulated genes in the *jouvence* overexpression condition versus the overexpressed genes in the *jouvence* depletion yielded 868 DEG in common (see Suppl. Table S8 for the list of genes, included in: Availability of Data and Materials). For example, among the 3098 overexpressed genes, DHRS2, SLC7A11, IL20RB, KLHDC7B, ROBO4, CDH15, CDLN2, that were up-regulated when *h-jou* was inhibited were down-regulated in the inverse condition of h-*jou* stably transfected cells (overexpression). For example, the dehydrogenase/reductase (SDR family) member 2 (DHRS2), known to inhibit cell growth and motility [41], was overexpressed in the siRNA cells. This result correlated with the observed phenotype after siRNA transfection, where the proliferation rate decreased significantly at 72 h post-transfection-1. Interestingly, these results suggest that snoRNA-*jouvence* gives rise to up- or down-regulation of a similar set of genes depending on its over- or under-expression. Taken together, these findings suggest that several genes are directly sensitive to the level of snoRNA-*jouvence*, and therefore their over or underexpression is unlikely to be a consequence of the deregulation of other genes.

## Discussion

We characterized a new snoRNA named *jouvence* in humans that had not previously been annotated in the genome; *jouvence* is localized on chromosome 11 in a large intron of the gene TEAD1. As previously reported [13], in addition to some tissues, *h-jou* is expressed, although weakly, in all tested well-established cell lines. To gain insights about the cellular role of *jouvence*, we first overexpressed *h-jou* in various cancerous and non-cancerous cell lines. Interestingly, the overexpression of *jouvence* led to significant increases in cell proliferation, yielding about twice the number of cells within a 1 week. More importantly, this effect was observed using two independent approaches to overexpress *h-jou*; first through a stably transfected-plasmid and second through a transduction with a lentivirus vector. This second result obtained with an integrative lentivirus suggests that snoRNA can be successfully carried by lentivirus. Inversely, the decrease (knockdown) of *h-jou* by transiently transfecting specific siRNA, led to the opposite phenotype characterized by rapid decrease of cell proliferation.

To further investigate the role of *h-jou*, and more particularly its molecular mechanism, we performed a transcriptomic analysis (RNA-Seq). The overexpression of *h-jou* led to up- and down-regulation of many genes. Among them, several deregulated genes, including Twist, SNAIL/SMUG/SMUC, ZEB, and fibronectin, suggest a dedifferentiation signature of the cell (Fig. 7). Therefore, it is tempting to speculate that this cellular effect could resemble, at least in part, a rejuvenation of the cells [42], an effect that remains to be experimentally demonstrated.

By contrast, decreasing *h-jou* through siRNA knockdown yielded an opposite cellular phenotype characterized by a striking decrease of cell RNA-Seq showed that several genes were deregulated, both up and down, while bioinformatics analysis revealed that the main affected KEGG pathways were, in addition to the metabolic pathway, the ribosome biogenesis and the spliceosome. Although *h-jou* harbours a non-canonical secondary structure of H/ACA box snoRNA, these results fit with the role of an H/ACA box snoRNA, known to be involved in the modification of rRNA [1–4], which consequently might affect the regulation of ribosome biogenesis. More precisely, according to the KEGG pathways, 99/138 genes of the ribosome pathway were affected, and among them, 91/99 were downregulated. For the spliceosome pathway, 90/134 genes were affected, and among them, 80/90 were downregulated. The metabolic pathway had 451/1243 genes affected, with 283/451 downregulated and 168/451 upregulated. Taken together, these results suggest a clear and an almost complete breakdown of the ribosome and the spliceosome pathways, which consequently could explain the decrease in proliferation of the cells, because protein synthesis might be strongly affected. In this context, it has already been described that repressing or perturbing ribosome biogenesis decreases the growth of the cells [43], and/or can even drive cells into senescence [44].

In the EMT process, epithelial cells acquire mesenchymal stem cells properties by losing cell-cell adhesion. Consequently, they become migratory and invasive [19, 20, 22]. The cancer cell lines with mesenchymal phenotype are characterized by under-expression of different genes such as claudins, cadherins, and occludins, among others. In addition, genes such as Fibronectin, and Jagged 1 are overexpressed, along with the three main EMT transcriptional factors: SNAIL/SLUG/SMUC, Twist and ZEB [18]. These factors can act together to induce EMT. Human SNAIL1 (SNAI1) protein encoded by SNAI1/SNA gene represses the transcription of the E-cadherin/CDH1 gene (Fig. 7). Human SNAIL2 (SNAI2) protein, encoded by the SNAI2/SLUG gene, induces the first phase of EMT, including desmosome dissociation, cell spreading, and initiation of cell separation [18]. Twist1 activates other EMT-inducing transcription factors to suppress E-cadherin and promote EMT and tumour metastasis. Here, in the *jouvence* overexpression context, several key genes involved in EMT, including TWIST, SNAIL, ZEB, and fibronectin, were upregulated, while some of their target genes such as claudin, E-cadherin, annexin, and α-catenin were downregulated (Fig. 7) [20, 22, 45, 46]. In brief, the overexpression of *h-jou* appears to be sufficient to re-orientate cells toward dedifferentiation, although HCT116 cells (an adenocarcinoma line from the gut) are originally cancerous cells. Nevertheless, the overexpression of *h-jou* appears to superimpose a signature of dedifferentiation. Alternatively, at the stage of this study, we could not exclude that this EMT process could rather be interpreted as a step toward the acquisition of a CSC phenotype. Indeed, a growing body of evidence suggests that snoRNAs may also play a role in CSC [12]. For example, ALDH1 has been demonstrated to be a CSC marker [47]. Here, several deregulated genes could also suggest a CSC mechanism, as few ALDH1-subunits, including ALDH1A3, ALDH1B1, and ALDH1L2, were affected. Other genes such as SOX4, SOX8, CD44, MSI-2 and EPCAM, which are also deregulated in *h-jou* depletion, have also been considered as potential CSC markers [48] (Fig. 7). Further experiments will be required to distinguish a CSC trend from an EMT dedifferentiation trend. Nonetheless, we have shown here that the overexpression of a single and short non-coding RNA of 159 bases, the snoRNA-*jouvence*, is sufficient to putatively reorient cells toward stemness.

According to its primary sequence, *jouvence* look-likes a snoRNA of type H/ACA box, generally known to perform pseudouridylation [1]. Pseudouridylation is a post-transcriptional isomerization reaction that converts uridine to pseudouridine (Ψ) [49]. The latter is present in several different types of RNAs, including coding and noncoding RNAs [5, 50]. Ψ is particularly concentrated in rRNA, and it plays an important role in protein translation, as well as in spliceosome snRNAs, in which it is involved in pre-mRNA splicing. Here, disrupting levels of *h-jou*, either by overexpression or by decreasing its expression, leads to the deregulation of quite a huge number of genes (around 6000: approximately half up and half down). Interestingly, among them, about 1000 genes (868 and 1102 respectively) are deregulated in the opposite direction when we compare *h-jou* overexpression with its depletion by siRNA (or vice-versa), suggesting that expression levels of these genes follow perfectly the level of *jou*, and consequently that they are very likely directly regulated by snoRNA-*jouvence*. In contrast, this suggests that the remaining deregulated genes (~ 5000) are rather likely a consequence of the deregulation of these “primary” 1000 genes, or in another words, it is a secondary effect. Further experiments will be required to decipher the precise molecular mechanisms of *jouvence* that lead to the deregulation of these several genes, either due to transcription or translation modifications, chromatin remodelling, RNA stability, or another mechanism.

In addition to the well-known canonical function of the snoRNA in various RNA modifications, more recently, some snoRNAs have been shown to be involved in stress responses and metabolic homeostasis. For example, the snoRNAs U32a, U33, and U35a are mediators of oxidative stress induced by palmitate and hydrogen peroxide [51], while the same snoRNAs also appear to regulate systemic glucose metabolism [52]. In the same way, Brandis et al. [53] showed that the C/D box snoRNA U60 regulates intracellular cholesterol trafficking between the plasma membrane and the endoplasmic reticulum. Similarly, the snoRNA U17 regulates cellular cholesterol trafficking through the hypoxia-upregulated mitochondrial regulator (HUMMR) by acting on its target mRNA [54]. Here, the modification of levels of the snoRNA-*jouvence*, either its overexpression or its depletion, leads to a strong deregulation of metabolic pathways as revealed by KEGG analysis (about 400 genes). Interestingly, among them, 3-hydroxy-3-methylglutaryl-CoA reductase (HMGCR), one of the key limiting enzymes involved in cholesterol synthesis (which is also the target of statins, anti-cholesterol medications), is upregulated when *jouvence* is overexpressed. Inversely, it is importantly downregulated when *jouvence* is depleted, suggesting that this crucial gene, involved in the regulation of the cholesterol level, is likely directly regulated by *jouvence*.

In the last several years, various snoRNAs (both C/D and H/ACA boxes) have been shown to be involved in cancer [10, 11], including in tumour initiation, invasion, metastasis, and/or proliferative signalling (for a list of snoRNA involved in cancer, see Table 1 in Liang et al. [11]). For example, some snoRNAs have been associated with the p53 pathway, a well-known tumour suppressor that responds to various cellular stresses regulating the expression of several target genes involved in cell cycle arrest, apoptosis, and DNA repair [55]. Here, in HCT116 cells, the p53 gene itself was not directly deregulated; however, several of its regulators were affected. Another crucial gene involved in cancer and more particularly in ribosome biogenesis is myc [56]. As example, it has been reported that in breast cancer, myc increases the expression levels of fibrillarin, a small nucleolar ribonucleoprotein (snoRNP) component. Subsequently, this increases snoRNA biogenesis, which in turn induces p53 suppression [57]. Interestingly, in various model organisms, including *Caenorhabditis elegans* [58], *Drosophila* [59], and mouse [60], links between Myc, ribosome biogenesis, and lifespan have been established. Furthermore, in *Drosophila*, it has been proposed that snoRNAs are a novel class of biologically relevant Myc targets [61]. Here, in the RNA-Seq performed on HCT116 cells overexpressing *jouvence*, MYC and MYCL, as well as some of their multiple regulators/interactors, were upregulated. In addition, the knockdown of the snoRNA pathway genes induced cellular stress, which led to the accumulation of p53 and consequently promoted the binding to some ribosomal proteins [55]. Moreover, the depletion of snoRNAs such as U3 and U8 led to ribosome dysfunction [62]. Interestingly, ribosome biogenesis, a crucial cellular function, was strongly affected in *jouvence* depleted HCT116 cells. Many other signalling pathways have been associated with various snoRNAs, including the phosphoinositide 3-kinase (PI3K)-AKT, and the Wnt/β-catenin pathways. Also here, in *jouvence*-depleted cells, the β-catenin, and/or some of its regulators are deregulated. Finally, SNORD76, a C/D box snoRNA, has been shown to act as a tumour suppressor in glioblastoma [63], a similar effect observed with the depletion of *jouvence* in the U87-MG glioblastoma cell line.

RNA-Seq analysis of *Drosophila jouvence* performed in two different contexts, knockdown and overexpression, have been recently reported [13]. The comparison of the KEGG analysis between *Drosophila* and human cells has shown that the metabolic pathways are the main common pathways affected in *jouvence*-knockdown. These results suggest that *jouvence* function could be related to certain cellular systems conserved throughout evolution. However, in contrast, in the *jouvence*-knockdown context, the striking decrease in ribosome and spliceosome pathway components observed in humans were not observed in *Drosophila*, suggesting that not all the cellular systems are conserved. Nevertheless, these experiments have not been performed on the same cell population. In humans, they were performed in a cancer cell line (HCT116), while in *Drosophila*, they were performed on epithelial cells of the gut, which mainly consist of enterocytes.

## Conclusion

As previously suggested [10, 11], snoRNAs have the potential to be cancer biomarkers, and may even become major cancer therapeutic targets in the near future. In this regard, snoRNA-*jouvence* is surely another promising candidate.

## Methods

### Cell lines and culture conditions

Cancer cell lines were obtained from the American Type Culture Collection (Rockville, MD, USA) and were cultured according to the supplier’s instructions. Primary Human Umbilical Vein Endothelial Cells (HUVEC) isolated from the vein of the umbilical cord were obtained from Promocell (Germany). HCT116 cells were cultured in McCoy’s 5A medium supplemented with 10% Fetal Bovine Serum and 1% glutamine. hTERT-RPE1 and Caco2 cells were maintained in Dulbecco’s modified Eagle’s medium: nutrient mixture F-12 supplemented with 10% FBS and 1% glutamine. HEK293 and U87-MG cells were cultured in DMEM 1X medium supplemented with 10% of FBS and 1% glutamine. MCF7 and A549 cells were cultured in RPMI 1640 supplemented with 10% Fetal Bovine Serum and 1% glutamine. HUVECs were grown in Endothelial Cell Growth Medium 2 which is low-serum (2% V/V) media optimized for the cultivation of endothelial cells from large blood vessels. Cells were incubated at 37 °C and at 5% CO_2_.

### Stable cell transfections

In order to generate stable cell lines that overexpress the human snoRNA-*jouvence*, the *h-jou* was cloned in the pcDNA3.1 Zeo + plasmid (ThermoFisher Scientific, USA) between EcoRI sites, in 5′ - 3′ direction behind a T7 promoter. HCT116, Caco2 or HEK 293 cells were transfected with the empty plasmid, serving as a control or with the plasmid containing *h-jou* cloned sequence. Briefly, cells were plated in 12-well plates and allowed to grow 24 h to reach nearly 60 to 70% of confluence at the time of transfection. Cells were transfected with the corresponding plasmid, using the Lipofectamine 3000 (ThermoFisher Scientific, USA) at a ratio of 1:2 for the DNA:lipofectamine. Lipofectamine 3000, p3000 and the plasmids were previously diluted in OptiMEM 1X (Gibco) (a Reduced-Serum Medium). The medium was changed 24 h after the transfection, and cells were allowed to grow for another additional 24 h before being split to a lower concentration. Seventy-two hours post-transfection, cells were treated with Zeocine (ThermoFisher Scientific) (75 μg/mL, 200 μg/mL, and 150 μg/mL for the HCT116, Caco2 and HEK 293 cells respectively). The selection of stably transfected cells was conducted over a period of nearly 4 to 5 weeks with the addition of Zeocine every 3 to 4 days. Stable clones expressing either the empty plasmid or the plasmid with the human snoRNA were selected, verified by RT-qPCR (TaqMan) before their use in the different experiments.

### Lentivirus preparation and in-vitro infection (transduction)

Classical Lentiviral integrative vector expressing the snoRNA-*jouvence* placed downstream to the U6 promoter (pLV.U6.hsnoRNA-jouvence), and containing the puromycine selection marker was generated by Flash Therapeutics/Vectalys (Toulouse, France). Cell infections were carried out according to Flash Therapeutics/Vectalys recommendations. For the HCT116 cell line, two MOI have been tested (MOI-1 and MOI-10). Since MOI-10 gives good results and the highest snoRNA-jou expression level, we uses MOI-10 for all other cell lines. For all cell lines, a puromycine selection has been performed for 48 h. In such condition, all non-transduced cells were eliminated (performed in parallel on non-lentivirus transduced cells, as control).

### Transfection of siRNA

The effect of the knockdown of the snoRNA-*jouvence* on the HCT116 cells was assessed by the transfection of short interfering RNA (siRNA). First, two different silencer selected siRNAs (Lock-Nucleotid-Acid siRNA or LNA-siRNA, shortly named siRNA) were tested (siRNA-1, antisense sequence: 3′-UCCUCUGUCCACAAUAGCC-5′, Cat nb: 4399665), and (siRNA-2, antisense sequence: 3′-UCAAGACCAAUCACCAUGU-5′, Cat nb: 4399665), as well as a non-targeting siRNA-control used for the specificity of the knockdown (Cat nb: 4390843) (ThermoFisher Scientific, USA). These two siRNAs (as well as the siRNA-control) were independently transfected into the cells in a 12-wells format. Cell suspensions of 0,25 × 10^6^ HCT116 cells per well were directly transfected with the corresponding siRNA (reverse transfection) at a final concentration of 10 nM per well, using the Lipofectamine RNAiMAX transfection reagent (ThermoFisher Scientific, USA). Forty-eight hours after the first siRNA reverse transfection, a forward transfection was performed on the adherent cells (at the same concentration as the first reverse transfection). After 24 h, the medium was changed and cells were kept in complete medium. Then, the cells were counted at different time points (days post-infection), depending of the cell line and compared to the different controls. Cell pellets (for the RNA extraction and RNA-seq analysis) were made 48 h post-transfection-2 (96 h after the first reverse transfection). Conditions were performed in triplicates and the specific knockdown of the snoRNA-*jouvence* was validated by standard RT-qPCR (TaqMan).

### VicellXR counting

The proliferation rate of the stably transfected cells (empty plasmid cells and snoRNA overexpressing cells) was analysed by cell counting. Cells were seeded in 6-well plates (in triplicates), with the following number of cells per well: 0,475 X 10^6^ HCTT16 cells per well; 0,5 X 10^6^ Caco2 cells per well and 0,2 X 10^6^ cells per well for the HEK293 cells. The experiments were conducted over a period of 96 to 160 h. Briefly, the supernatant was harvested and cells washed with PBS 1X. Then, cells were trypsinized with 300 μl of 0.05% trypsin/EDTA (Gibco) per well. Trypsine was inactivated with 700 μL of the corresponding medium per well, and cells were then counted with the VicellXR using trypan blue to determine cell viability (Cell viability Analyzer, Beckman Coulter).

### CellTiter-Glo

A luminescent cell viability assay (CellTiter-Glo, Promega, USA) was assessed to determine the cell proliferation rate based on quantitation of the ATP, an indicator of metabolically active cells. Three thousand cells were plated in each well of a 96-well white plate with clear bottom (Costar 3610, Corning Incorporated, USA). Each cell condition was plated in 6 or 10 wells. Cells were allowed to adhere for 24 h, before counting. The homogeneous assay procedure involves addition of 100 μL of the CellTiter-Glo reagent directly to the cells cultured in 100 μL of their corresponding complete medium, before measuring the relative luminescence using a multiplate reader (POLAR Star Omega BMG LABTECH). The experiments were conducted over a week, with counting performed every 24 h for HCT116 and HEK293, and at 96 h for the lentivirus transduction.

### RNA extraction and reverse transcription (RT)

Total RNA from human cell lines were extracted using NucleoSpin RNA Plus columns (Macherey-Nagel, France), according to the manufacturer’s instructions. Extracted RNAs were verified for the absence of genomic DNA contamination, by performing a RT-PCR using the ribosomal gene RP49. Contaminated samples were therefore treated with RQ1 DNase (Promega, USA) and cleaned with the NucleoSpin RNA Clean-UP (Macherey-Nagel). Two micrograms of total RNA were used for the synthesis of cDNA with oligo-dT (used for Sybr Green RT-qPCR) or random primers (used for TaqMan RT-qPCR for the detection of the snoRNA expression). The M-MULV reverse transcriptase (Promega, USA) was used and the RT was performed in a final volume of 25 μL. The thermal cycling conditions were 37 °C for 50 min followed by 15 min at 70 °C. RNase H (Invitrogen, USA) was performed to digest RNA-DNA hybrids.

### RT-qPCR (real time quantitative polymerase chain reaction) on selected genes

The differential expression of the snoRNA and selected genes were analysed by real-time PCR (QuantStudio 3, Applied Biosystems, France). The expression of the snoRNA was detected with TaqMan customized probes (Applied Biosciences, Life Technologies) and the TaqMan Universal Master Mix II, no UNG (Applied Biosystems). All conditions were normalised to the GAPDH control gene. The Sybr Green RT-qPCR was performed for all the other genes with the Power UP SYBR-Green PCR Master Mix, according to the manufacturer’s instructions (Applied Biosystems). Primers were designed using the “Primer 3 Plus” software (the primer sequences will be provided upon request). All conditions were normalised to the RPLP0 control gene. The results were analysed using the 2-ΔΔCt method, and displayed as the fold change compared to the control gene.

### Transcriptomic analysis (RNA-seq)

The transcriptomic analysis (RNA-seq) was performed by Novogene (China). Briefly, total RNA was extracted and after sample quality control, libraries enriched for polyA RNAs, were generated and checked for quality. Then the libraries were sequenced on an Illumina Hiseq platform and 125 bp/150 bp paired-end reads were generated. The resulting data were controlled, and analyzed with bioinformatic tools. For all the computational tools analysis, common defaults parameter values were used. The reference genome and gene model annotation files were downloaded from genome website directly (see: Availability of Data and Materials). Bowtie v2.2.3 was used to build the index of the reference genome. TopHat v2.0.12. was used to align paired-end clean reads to the reference genome, because it can generate a database of splice junctions based on the gene model annotation file, yielding to a better mapping result than other non-splice mapping tools. For the quantification of gene expression level, HTSeq v0.6.1 was used to count the read numbers mapped to each gene. Then FPKM of each gene was calculated based on the length of the gene and read counts mapped to this gene. The expected number of Fragments Per Kilobase of transcript sequence per Millions base pairs sequenced (FPKM) is the commonly used method for estimating gene expression levels. Indeed, it takes in account the effect of sequencing depth and gene length for the read counts at the same time. Differential expression analysis of two conditions (three biological replicates per condition) was performed using the DESeq R package (1.18.0). DESeq uses a model based on the negative binomial distribution to determine differential expression in digital gene expression data. Then, the *P* values were adjusted using the Benjamini & Hochberg method. Corrected *P*-value of 0,05 and log2 (Fold-change) of 1 were set as the threshold for significant differential expression. The biological variation was eliminated (case with biological replicates), and the threshold was therefore normally set as p adjusted < 0,05. Gene Ontology (GO) enrichment analysis of differentially expressed genes was implemented by the GOseq R package, in which gene length bias was corrected. GO terms with corrected *p*-value less than 0,05 were considered significantly enriched by differential expressed genes. For the KEGG database [15, 16], KOBAS software was used to test the statistical enrichment of differential expression genes in KEGG pathways.

### Statistical analysis

All data were analysed statistically using one-tailed unpaired t-test, with GraphPad Prism™ software.

## Supplementary Information

**Additional file 1: Suppl. Figure 1.** Validation by RT-qPCR of few selected deregulated genes in *jou* overexpression or knockdown. **A)** RT-qPCR (SybGreen) results of the quantification of few selected genes in *jou* overexpressing HCT116 cells. Fold change comparing HCT116 transfected cells versus empty plasmid cells. As revealed by the RNA-Seq, the genes BCL2, CLDN9, CTNNAL, and HNF4A are downregulated, while the genes CTGF, HDAC5, IGF1R, SNAI3, IRS2, and SOX4 are upregulated. **B)** RT-qPCR (SybGreen) results of the quantification of few selected genes in *jou* knockdown. Fold change comparing HCT116 siRNA transfected cells versus non-transfected cells. FDFT1, HMGCR, PRPF, SRSF4 are downregulated, while CLDN2, DHRS2, ILORB, and SLC7A11 genes are upregulated (*n* = 2). The RT-qPCR confirms the deregulation of those genes revealed by RNA-Seq.

**Additional file 2: Table S1.** HCT-jou-overexpression_DEG upregulated genes.

**Additional file 3: Table S2.** HCT-jou-overexpression_DEG downregulated genes.

**Additional file 4: Table S3.** HCT-jou-overexpression_KEGG enrichment pathways.

**Additional file 5: Table S4.** HCT-siRNA knockdown_DEG upregulated genes.

**Additional file 6: Table S5.** HCT-siRNA knockdown_DEG downregulated genes.

**Additional file 7: Table S6.** HCT-siRNA knockdown_KEGG enrichment pathways.

**Additional file 8: Table S7.** jou-overexpression: genes Up versus siRNA: genes down (1102 genes).

**Additional file 9: Table S8.** jou-overexpression: genes Down versus siRNA: genes Up (868 genes).

## Data Availability

The datasets generated during the current study are available in the NCBI Sequence Read Archive (SRA) with the BioSample accession number: PRJNA670938. Moreover, all the analyzed RNA-Seq data files are available in the Supplementary Information.

## Abbreviations

CSC: Cancer stem cells
DEG: Differentially expressed genes
EMT: Epithelial-mesenchymal transition
*h-jou*: Human *jouvence*
KEGG: Kyoto Encyclopedia of Genes and Genomes
MET: Mesenchymal-Epithelial-Transition
MOI: Multiplicities of infection
rRNA: Ribosomal RNA
snoRNAs: Small nucleolar RNAs

## References

[1] Kiss T (2002). Small nucleolar RNAs: an abundant group of noncoding RNAs with diverse cellular functions. Cell..

[2] Gardner PP, Bateman A, Poole AM (2010). SnoPatrol: how many snoRNA genes are there?. J Biol.

[3] Ye K (2007). H/ACA guide RNAs, proteins and complexes. Curr Opin Struct Biol.

[4] Kiss T, Fayet-Lebaron E, Jády BE (2010). Box H/ACA small ribonucleoproteins. Mol Cell.

[5] McMahon M, Contreras A, Ruggero D (2015). Small RNAs with big implications: new insights into H/ACA snoRNA function and their role in human disease. Wiley Interdiscip Rev RNA.

[6] Mitchell JR, Wood E, Collins K (1999). A telomerase component is defective in the human disease dyskeratosis congenita. Nature..

[7] Vulliamy T, Marrone A, Goldman F, Dearlove A, Bessler M, Mason PJ (2001). The RNA component of telomerase is mutated in autosomal dominant dyskeratosis congenita. Nature..

[8] Cavaillé J, Buiting K, Kiefmann M, Lalande M, Brannan CI, Horsthemke B (2000). Identification of brain-specific and imprinted small nucleolar RNA genes exhibiting an unusual genomic organization. Proc Natl Acad Sci U S A.

[9] Kishore S, Stamm S (2006). The snoRNA HBII-52 regulates alternative splicing of the serotonin receptor 2C. Science..

[10] Mannoor K, Liao J, Jiang F (1826). Small nucleolar RNAs in cancer. Biochim Biophys Acta.

[11] Liang J, Wen J, Huang Z, Chen XP, Zhang BX, Chu L (2019). Small Nucleolar RNAs: insight into their function in Cancer. Front Oncol.

[12] Batlle E, Clevers H (2017). Cancer stem cells revisited. Nat Med.

[13] Soulé S, Mellottée L, Arab A, Chen C, Martin JR (2020). Jouvence a small nucleolar RNA required in the gut extends lifespan in Drosophila. Nat Commun.

[14] Taoka M, Nobe Y, Yamaki Y, Sato K, Ishikawa H, Izumikawa K (2018). Landscape of the complete RNA chemical modifications in the human 80S ribosome. Nucleic Acids Res.

[15] Kanehisa M, Furumichi M, Tanabe M, Sato Y, Morishima K (2017). KEGG: new perspectives on genomes, pathways, diseases and drugs. Nucleic Acids Res.

[16] Kanehisa M, Sato Y, Furumichi M, Morishima K, Tanabe M (2018). New approach for understanding genome variations in KEGG. Nucleic Acids Res.

[17] Takahashi K, Yamanaka S (2006). Induction of pluripotent stem cells from mouse embryonic and adult fibroblast cultures by defined factors. Cell..

[18] De Craene B, Berx G (2013). Regulatory networks defining EMT during cancer initiation and progression. Nat Rev Cancer.

[19] Pastushenko I, Blanpain C (2019). EMT transition states during tumor progression and metastasis. Trends Cell Biol.

[20] Santos F, Moreira C, Nóbrega-Pereira S, Bernardes de Jesus B (2019). New Insights into the Role of Epithelial-Mesenchymal Transition during Aging. Int J Mol Sci.

[21] Hwang W, Chiu YF, Kuo MH, Lee KL, Lee AC, Yu CC (2017). Expression of neuroendocrine factor VGF in lung Cancer cells confers resistance to EGFR kinase inhibitors and triggers epithelial-to-Mesenchymal transition. Cancer Res.

[22] Thiery JP, Acloque H, Huang RY, Nieto MA (2009). Epithelial-mesenchymal transitions in development and disease. Cell..

[23] Sosa MS, Parikh F, Maia AG, Estrada Y, Bosch A, Bragado P (2015). NR2F1 controls tumour cell dormancy via SOX9- and RARβ-driven quiescence programmes. Nat Commun.

[24] Kim IG, Lee JH, Kim SY, Hwang HM, Kim TR, Cho EW (2018). Hypoxia-inducible transgelin 2 selects epithelial-to-mesenchymal transition and γ-radiation-resistant subtypes by focal adhesion kinase-associated insulin-like growth factor 1 receptor activation in non-small-cell lung cancer cells. Cancer Sci.

[25] Reichenbach B, Classon J, Aida T, Tanaka K, Genander M, Göritz C. Glutamate transporter Slc1a3 mediates inter-niche stem cell activation during skin growth. EMBO J. 2018 May 2;37(9):e98280. 10.15252/embj.201798280. 10.15252/embj.201798280 PMC5920238 29615452

[26] Shan LN, Song YG, Su D, Liu YL, Shi XB, Lu SJ (2015). Early growth response Protein-1 involves in transforming growth factor-β1 induced epithelial-Mesenchymal transition and inhibits migration of non-small-cell lung Cancer cells. Asian Pac J Cancer Prev.

[27] Wang H, Zhou Y, Yu D, Zhu H (2016). Klf2 contributes to the stemness and self-renewal of human bone marrow stromal cells. Cytotechnology..

[28] Korpal M, El BJ, Buffa FM, Ibrahim T, Blanco MA, Celià-Terrassa T (2011). Direct targeting of Sec23a by miR-200s influences cancer cell secretome and promotes metastatic colonization. Nat Med.

[29] Sekiya S, Suzuki A (2011). Direct conversion of mouse fibroblasts to hepatocyte-like cells by defined factors. Nature..

[30] Hatta M, Miyake Y, Uchida K, Yamazaki J (2018). Keratin 13 gene is epigenetically suppressed during transforming growth factor-β1-induced epithelial-mesenchymal transition in a human keratinocyte cell line. Biochem Biophys Res Commun.

[31] Tan M, Liu C, Huang W, Deng L, Qin X, Xiang Y (2018). CTNNAL1 inhibits ozone-induced epithelial-mesenchymal transition in human bronchial epithelial cells. Exp Physiol.

[32] Hosono Y, Yamaguchi T, Mizutani E, Yanagisawa K, Arima C, Tomida S (2012). MYBPH, a transcriptional target of TTF-1, inhibits ROCK1, and reduces cell motility and metastasis. EMBO J.

[33] Gon Y, Maruoka S, Kishi H, Kozu Y, Kazumichi K, Nomura Y (2017). NDRG1 is important to maintain the integrity of airway epithelial barrier through claudin-9 expression. Cell Biol Int.

[34] Lee MJ, Yu GR, Yoo HJ, Kim JH, Yoon BI, Choi YK (2009). ANXA8 down-regulation by EGF-FOXO4 signaling is involved in cell scattering and tumor metastasis of cholangiocarcinoma. Gastroenterology..

[35] Faura Tellez G, Vandepoele K, Brouwer U, Koning H, Elderman RM, Hackett TL (2015). Protocadherin-1 binds to SMAD3 and suppresses TGF-β1-induced gene transcription. Am J Physiol Lung Cell Mol Physiol.

[36] Peng X, Liu G, Peng H, Chen A, Zha L, Wang Z (2017). SOX4 contributes to TGF-β-induced epithelial-mesenchymal transition and stem cell characteristics of gastric cancer cells. Genes Dis.

[37] Xie SL, Fan S, Zhang SY, Chen WX, Li QX, Pan GK (2018). SOX8 regulates cancer stem-like properties and cisplatin-induced EMT in tongue squamous cell carcinoma by acting on the Wnt/β-catenin pathway. Int J Cancer.

[38] Mokrowiecka A, Veits L, Falkeis C, Musial J, Kordek R, Lochowski M (2017). Expression profiles of cancer stem cell markers: CD133, CD44, Musashi-1 and EpCAM in the cardiac mucosa-Barrett's esophagus-early esophageal adenocarcinoma-advanced esophageal adenocarcinoma sequence. Pathol Res Pract.

[39] Jeong G, Bae H, Jeong D, Ham J, Park S, Kim HW (2018). A Kelch domain-containing KLHDC7B and a long non-coding RNA ST8SIA6-AS1 act oppositely on breast cancer cell proliferation via the interferon signaling pathway. Sci Rep.

[40] Enomoto S, Mitsui K, Kawamura T, Iwanari H, Daigo K, Horiuchi K (2016). Suppression of Slit2/Robo1 mediated HUVEC migration by Robo4. Biochem Biophys Res Commun.

[41] Zhou Y, Wang L, Ban X, Zeng T, Zhu Y, Li M (2018). DHRS2 inhibits cell growth and motility in esophageal squamous cell carcinoma. Oncogene..

[42] Rando TA, Chang HY (2012). Aging, rejuvenation, and epigenetic reprogramming: resetting the aging clock. Cell..

[43] Hald ØH, Olsen L, Gallo-Oller G, Elfman LHM, Løkke C, Kogner P (2019). Inhibitors of ribosome biogenesis repress the growth of MYCN-amplified neuroblastoma. Oncogene..

[44] Nishimura K, Kumazawa T, Kuroda T, Katagiri N, Tsuchiya M, Goto N (2015). Perturbation of ribosome biogenesis drives cells into senescence through 5S RNP-mediated p53 activation. Cell Rep.

[45] Jiang Y, Cao W, Wu K, Qin X, Wang X, Li Y (2019). LncRNA LINC00460 promotes EMT in head and neck squamous cell carcinoma by facilitating peroxiredoxin-1 into the nucleus. J Exp Clin Cancer Res.

[46] Lamouille S, Xu J, Derynck R (2014). Molecular mechanisms of epithelial-mesenchymal transition. Nat Rev Mol Cell Biol.

[47] Mannoor K, Shen J, Liao J, Liu Z, Jiang F (2014). Small nucleolar RNA signatures of lung tumor-initiating cells. Mol Cancer.

[48] Gopalan V, Islam F, Lam AK (2018). Surface markers for the identification of Cancer stem cells. Methods Mol Biol.

[49] Ofengand J, Bakin A (1997). Mapping to nucleotide resolution of pseudouridine residues in large subunit ribosomal RNAs from representative eukaryotes, prokaryotes, archaebacteria, mitochondria and chloroplasts. J Mol Biol.

[50] Adachi H, De Zoysa MD, Yu YT (1862). Post-transcriptional pseudouridylation in mRNA as well as in some major types of noncoding RNAs. Biochim Biophys Acta Gene Regul Mech.

[51] Michel CI, Holley CL, Scruggs BS, Sidhu R, Brookheart RT, Listenberger LL (2011). Small nucleolar RNAs U32a, U33, and U35a are critical mediators of metabolic stress. Cell Metab.

[52] Lee J, Harris AN, Holley CL, Mahadevan J, Pyles KD, Lavagnino Z (2016). Rpl13a small nucleolar RNAs regulate systemic glucose metabolism. J Clin Invest.

[53] Brandis KA, Gale S, Jinn S, Langmade SJ, Dudley-Rucker N, Jiang H (2013). Box C/D small nucleolar RNA (snoRNA) U60 regulates intracellular cholesterol trafficking. J Biol Chem.

[54] Jinn S, Brandis KA, Ren A, Chacko A, Dudley-Rucker N, Gale SE (2015). snoRNA U17 regulates cellular cholesterol trafficking. Cell Metab.

[55] Joerger AC, Fersht AR (2016). The p53 pathway: origins, inactivation in Cancer, and emerging therapeutic approaches. Annu Rev Biochem.

[56] van Riggelen J, Yetil A, Felsher DW (2010). MYC as a regulator of ribosome biogenesis and protein synthesis. Nat Rev Cancer.

[57] Su H, Xu T, Ganapathy S, Shadfan M, Long M, Huang TH (2014). Elevated snoRNA biogenesis is essential in breast cancer. Oncogene..

[58] Johnson DW, Llop JR, Farrell SF, Yuan J, Stolzenburg LR, Samuelson AV. The Caenorhabditis elegans Myc-Mondo/Mad complexes integrate diverse longevity signals. PLoS Genet. 2014;10(4):e1004278. 10.1371/journal.pgen.1004278 PMC3974684 24699255

[59] Greer C, Lee M, Westerhof M, Milholland B, Spokony R, Vijg J, et al. Myc-dependent genome instability and lifespan in Drosophila. PLoS One. 2013;8:e74641. 10.1371/journal.pone.0074641 PMC3765364 24040302

[60] Hofmann JW, Zhao X, De Cecco M, Peterson AL, Pagliaroli L, Manivannan J, et al. Reduced expression of MYC increases longevity and enhances healthspan. Cell. 2015;160:477–88. 10.1016/j.cell.2014.12.016 PMC4624921 25619689

[61] Herter EK, Stauch M, Gallant M, Wolf E, Raabe T, Gallant P. snoRNAs are a novel class of biologically relevant Myc targets. BMC Biol. 2015;13:25. 10.1186/s12915-015-0132-6 PMC4430873 25888729

[62] Langhendries JL, Nicolas E, Doumont G, Goldman S, Lafontaine DL. The human box C/D snoRNAs U3 and U8 are required for pre-rRNA processing and tumorigenesis. Oncotarget. 2016;7:59519–34. 10.18632/oncotarget.11148 PMC5312328 27517747

[63] Chen L, Han L, Wei J, Zhang K, Shi Z, Duan R, et al. SNORD76, a box C/D snoRNA, acts as a tumor suppressor in glioblastoma. Sci Rep. 2015;5:8588. 10.1038/srep08588. 10.1038/srep08588 PMC5390076 25715874

