# Supplementary figures and images for: jouvence, a new human snoRNA involved in the control of cell proliferation
Source: BMC Genomics. 2020 Nov 23;21:817. doi: 10.1186/s12864-020-07197-3 (PMC7682050; doi:10.1186/s12864-020-07197-3)

## Slide 1
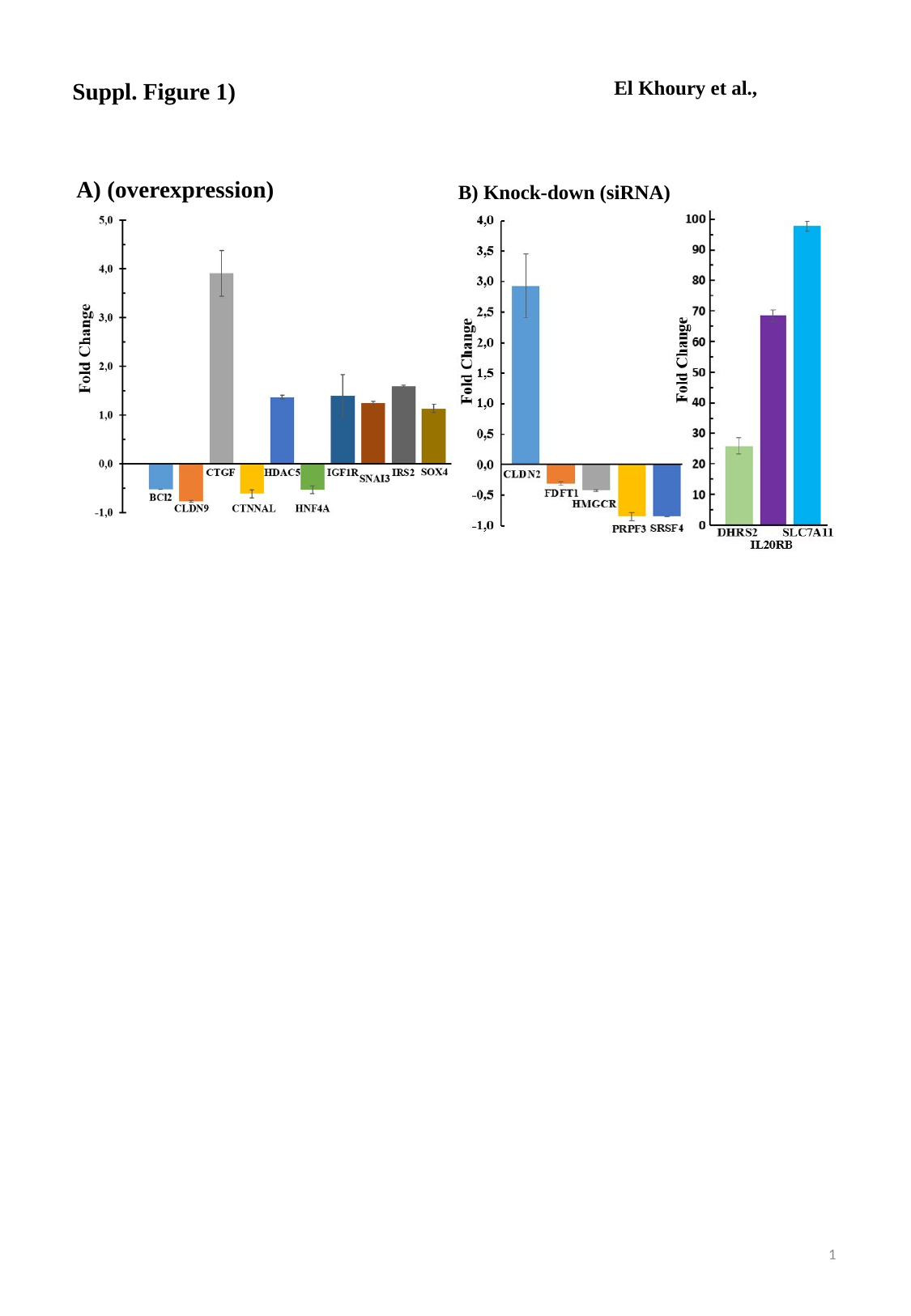

Suppl. Figure 1)
El Khoury et al.,
A) (overexpression)
B) Knock-down (siRNA)
1

Supplement: Supplementary file 1 — Additional file 1: Suppl. Figure 1. Validation by RT-qPCR of few selected deregulated genes in jou overexpression or knockdown. A) RT-qPCR (SybGreen) results of the quantification of few selected genes in jou overexpressing HCT116 cells. Fold change comparing HCT116 transfected cells versus empty plasmid cells. As revealed by the RNA-Seq, the genes BCL2, CLDN9, CTNNAL, and HNF4A are downregulated, while the genes CTGF, HDAC5, IGF1R, SNAI3, IRS2, and SOX4 are upregulated. B) RT-qPCR (SybGreen) results of the quantification of few selected genes in jou knockdown. Fold change comparing HCT116 siRNA transfected cells versus non-transfected cells. FDFT1, HMGCR, PRPF, SRSF4 are downregulated, while CLDN2, DHRS2, ILORB, and SLC7A11 genes are upregulated (n = 2). The RT-qPCR confirms the deregulation of those genes revealed by RNA-Seq. [file 12864_2020_7197_MOESM1_ESM.pptx]
